# Multimer recognition and secretion by the non-classical secretion pathway in *Bacillus subtilis*

**DOI:** 10.1038/srep44023

**Published:** 2017-03-09

**Authors:** Liuqun Zhao, Jingqi Chen, Jibin Sun, Dawei Zhang

**Affiliations:** 1 Tianjin Institute of Industrial Biotechnology, Chinese Academy of Sciences, Tianjin 300308, P. R. China; 2 Key Laboratory of Systems Microbial Biotechnology, Chinese Academy of Sciences, Tianjin 300308, P. R. China; 3 National Engineering Laboratory for Industrial Enzymes, Tianjin 300308, P. R. China

## Abstract

Non-classical protein secretion in bacteria is a common phenomenon. However, the selection principle for non-classical secretion pathways remains unclear. Here, our experimental data, to our knowledge, are the first to show that folded multimeric proteins can be recognized and excreted by a non-classical secretion pathway in *Bacillus subtilis*. We explored the secretion pattern of a typical cytoplasmic protein D-psicose 3-epimerase from *Ruminococcus sp*. 5_1_39BFAA (RDPE), and showed that its non-classical secretion is not simply due to cell lysis. Analysis of truncation variants revealed that the C- and N-terminus, and two hydrophobic domains, are required for structural stability and non-classical secretion of RDPE. Alanine scanning mutagenesis of the hydrophobic segments of RDPE revealed that hydrophobic residues mediated the equilibrium between its folded and unfolded forms. Reporter mCherry and GFP fusions with RDPE regions show that its secretion requires an intact tetrameric protein complex. Using cross-linked tetramers, we show that folded tetrameric RDPE can be secreted as a single unit. Finally, we provide evidence that the non-classical secretion pathway has a strong preference for multimeric substrates, which accumulate at the poles and septum region. Altogether, these data show that a multimer recognition mechanism is likely applicable across the non-classical secretion pathway.

Protein secretion plays an important role in the physiological process of bacterial cells and their communication with the environment. Cells can export proteins from the cytoplasm via multiple secretion mechanisms, among which two conserved systems account for the majority of protein export: the general secretion (Sec) pathway and the twin-arginine transport (Tat) pathway^1^,^2^. The Sec system translocates unfolded proteins, and the Tat system secretes folded proteins across the cytoplasmic membrane and the cell wall^3^,^4^. In addition to these classical pathways, there are numerous specific secretion machineries that export certain proteins into the extracellular milieu in bacteria. Some excreted proteins or antimicrobial peptides that are inherently incompatible with the Sec or Tat pathway might use the ABC export systems that facilitate their secretion^5^. The vesicle-mediated export pathway can also export some specific proteins^6^. Recently, the ESX secretion system, which is responsible for secretion of small proteins containing a conserved WXG motif, has been characterized in various bacteria^7^,^8^.

While many proteins are excreted into the extracellular space with known secretion signals, a growing number of typical cytoplasmic proteins lacking any signal peptides or secretion motifs are found in the supernatant of several bacteria, which are termed non-classically secreted proteins^9^. Vincent Fischetti’s group found that glyceraldehyde-3-phosphate dehydrogenase (GAPDH) is present on the cell surface of pathogenic streptococci species and in the extracellular milieu of some bacteria, fungi and even protozoans^10^,^11^. In addition, several cytoplasmic proteins have been found on the cell surface or in the culture supernatant, such as fibrinogen-binding protein A and enolase of *Listeria monocytogenes*^12^,^13^, glucose-6-phosphate isomerase of *Streptococcus agalactiae*^14^ or glutamine synthetase of *Mycobacterium tuberculosis*^15^. Proteome analysis of *Bacillus subtilis* extracellular proteins revealed that approximately 50% of these proteins were not projected in previous genome-based predictions. This is mainly due to difficulties in the prediction of extracellular proteins that contain retention signals and lack signal peptides (including cytoplasmic proteins)^16^,^17^,^18^. Using two-dimensional polyacrylamide gel electrophoresis (2D-PAGE), 17 cytoplasmic proteins that contain no typical signal peptides were identified in the extracellular milieu in *B. subtilis*^19^. Wang *et al*. listed 45 common non-classically secreted proteins identified in various bacteria^9^, hinting that non-classical secretion is a universal phenomenon in bacteria.

Some non-classically secreted proteins are “moonlighting proteins” that display two different functions in the cytoplasmic and extracellular environments^20^. The moonlighting activities of these cytoplasmic proteins play a role in bacterial virulence in pathogenic bacteria^21^. The extracellular function and localization of these non-classically secreted proteins have been analysed in various bacteria; however, the mechanism of non-classical secretion pathways has not yet been identified. Recently, 22 typical cytoplasmic proteins were found in the secretomeof *Staphylococcus aureus*, whereas the most abundant proteins were absent, suggesting a selection principle for non-classical protein export^22^. Other experimental evidence also strongly supports this issue. In *Escherichia coli*, the activity of the glycolytic enzyme enolase is involved in the binding of its substrate 2-phosphoglycerate (2-PG) to the active site Lys341. Replacement of Lys341 with other amino acids not only prevented the auto-modification but also halted the secretion of enolase^23^. Interestingly, a similar result was obtained with the enolase from *B. subtilis*, which possesses a hydrophobic α-helical domain that contributes to its secretion^24^. Consequently, a specific selection process is involved in non-classical protein secretion in bacteria.

In this context, many researchers have focused their attention on determining the secretion mechanism for non-classically secreted proteins. The long-held assumption is that these proteins are simply released by cell lysis^25^. However, an increasing number of studies imply that non-classical protein secretion is the result of an as-yet unknown mechanism^20^,^25^. Another system is membrane vesicles (MVs) formation, which is known to release cytoplasmic proteins from Gram-positive bacteria^26^. The auxiliary protein secretion system (SecA2), as a specialized secretion system, may contribute to the excretion of cytoplasmic proteins in *M. tuberculosis*^27^. It is unknown whether some of these cytoplasmic proteins are eventually exported from one of these secretion systems^20^. Thus, no generally applicable mechanism involved in the excretion of cytoplasmic proteins has been reported^28^.

In bacteria, non-classical protein secretion is not well understood and, many unanswered questions remain^28^. Here, we addressed the question of whether a specific selection process is involved in the excretion of cytoplasmic proteins. In this report, we analysed the excretion pattern of a cytoplasmic protein D-psicose 3-epimerase, from *Ruminococcus sp*. 5_1_39BFAA (RDPE) in *B. subtilis*. We demonstrate that folded RDPE is likely exported as a single unit by a non-classical secretion system. Moreover, various multimeric cytoplasmic proteins were translocated through the cytoplasmic membrane at the poles and septum region via a non-classical secretion pathway. Together, our results show that the non-classical secretion system requires an as-yet unknown recognition signal formed by folded polypeptides for secretion and could export an intact multimeric protein complex.

## Results

### Secretion of cytoplasmic protein RDPE is not simply due to cell lysis

**Figure 1:**
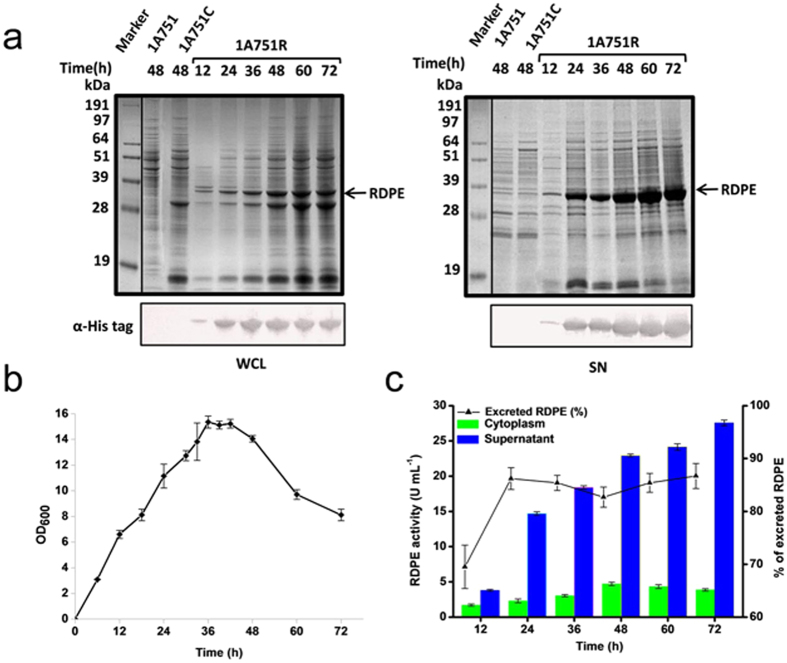
Secretion of RDPE in *B. subtilis*. (**a**) SDS-PAGE and western blot analysis of the whole cell lysates (WCL) and culture supernatant (SN) fractions of 1A751R strain expressing RDPE protein. The parent strain 1A751 and 1A751C harboring empty vector pMA5 are regarded as negative controls. The band of RDPE (33 kDa) is labeled with an arrow. (**b**) Growth curve of *B. subtilis* strain 1A751R. Error bars are SDs from three independent experiments. (**c**) The relative amount of RDPE was followed over time by enzyme activity measurement. Error bars are SDs from three independent experiments.

We previously reported that RDPE, which converts D-fructose into D-psicose from *Ruminococcus sp*. 5_1_39BFAA, can be secreted into the medium from *B. subtilis* without a typical signal peptide. Moreover, we verified that RDPE secretion is independent on Sec and Tat pathway^29^. By studying the time course of RDPE excretion, it is possible to observe whether the excretion level is constant or growth phase-dependent. For this purpose, we constructed a multicopy pMA5R plasmid containing the *rdpe* gene under the control of the strong constitutive promoter *P*_*HpaII*_. This plasmid was transformed into *B. subtilis* 1A751 cells for overexpression of RDPE, resulting in the recombinant strain 1A751R. Therefore, we followed RDPE excretion during the growth phase by SDS-PAGE and western blot analysis (Fig. 1a,b). When examining the content of RDPE in the culture medium over time, there was already a visible band seen after 12 h of growth whose intensity steadily increased up to 72 h. In contrast, the intracellular levels of RDPE reached a plateau after 48 h of growth and, gradually decreased through the decline phase. We wondered how much of the cytoplasmic protein was secreted. We therefore compared the enzyme activity of RDPE in the cytoplasm with that in the culture supernatant over time (Fig. 1c). After entry into the mid-exponential growth phase, approximately 83–87% of RDPE was found in the supernatant. The relative amount of RDPE increased slightly during the decline phase, suggesting that cell lysis appears to play a role in its secretion. These results indicate that the secretion of RDPE occurs in the early-exponential phase and that the secretion efficiency was nearly constant during the growth phase, even though extensive cell lysis occurred in the late-stationary growth phase.

**Figure 2:**
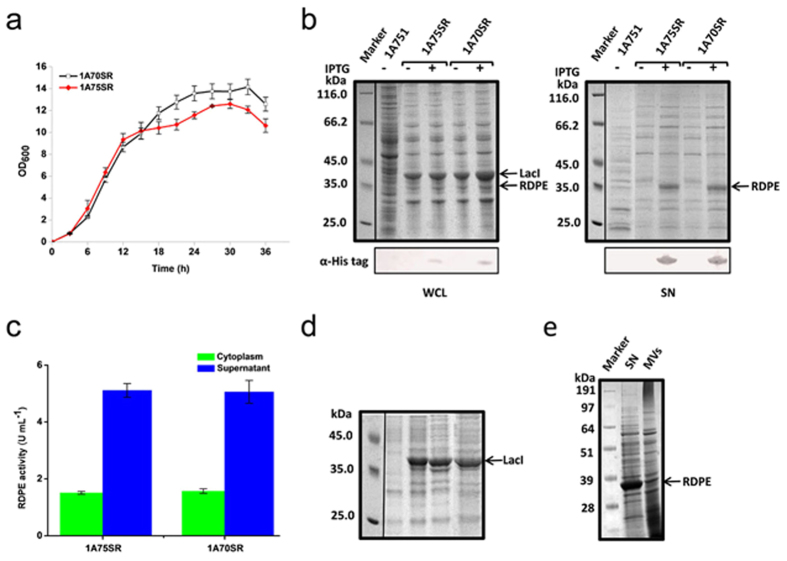
Excretion of RDPE is not simply due to cell lysis. (**a**) Growth curve of *B. subtilis* strains 1A75SR and 1A70SR. Error bars are SDs from three independent experiments. (**b**) Inducible expression of RDPE in *B. subtilis* 1A751 and a *lytC lytD* double deletion strain 1A700. When the culture reached an optical density at 600 nm of 0.8, IPTG was added to a final concentration of 1 mM to induce RDPE expression. After 24 h, sample fractions were harvested and analysed by SDS-PAGE and western blot. The parent strain 1A751, 1A75SR (expressing RDPE) and 1A70SR (lacking *lytC lytD* and expressing RDPE) without induced by IPTG are regarded as negative controls. (**c**) Enzyme activity analysis of RDPE from the strains 1A75SR and 1A70SR in the cytoplasm and culture supernatant with 24 h incubation. Error bars are SDs from three independent experiments. (**d**) The stability of LacI protein in the cell supernatant. The soluble cell extract was added to the culture after 30 h of growth. The samples were analysed by SDS-PAGE. The control line contains control sample without addition of crude extracts. The remaining lanes contain samples at different times (0, 60 and 120 min) after addition of whole-cell extracts. (**e**) SDS-PAGE analysis of RDPE in culture supernatant (SN) and membrane vesicles (MVs) of *B. subtilis* 1A751R. The arrows indicate the location of target proteins.

To determine whether the secretion of RDPE was caused by cell lysis, we generated a mutant strain 1A700 with deficiency in two genes *lytC* and *lytD*. As two major autolysin genes, *lytC* and *lytD*, are responsible for autolytic activity, mutants of *lytC* or *lytD* are resistant to cell lysis^30^. We also overexpressed the RDPE protein in the WB600 strain, which is deficient in six extracellular proteases^31^. The expression and secretion levels were similar to those of the strain 1A751R (Supplementary Fig. S1). The data showed that the RDPE protein possessed high extracellular stability. As cytoplasmic proteins may be released by cell lysis due to their high expression and extracellular stability^32^, to achieve low expression levels of RDPE, the strong promoter *P*_*HpaII*_ was replaced by a weak IPTG-inducible promoter *P*_*spac*_, resulting in pMASR. This recombinant plasmid was transformed into the parental strain *B. subtilis* 1A751 and the mutant strain 1A700 cells for overexpression, resulting in strains 1A75SR and 1A70SR, respectively. As expected, the strain 1A70SR exhibited less cell lysis than the strain 1A75SR (Fig. 2a). To reduce the effect of cell lysis on secretion level, all samples used for analysis were collected at 24 h. We observed very low expression levels of RDPE protein in the cells, but a detectable level in the culture supernatant. Furthermore, there were similar levels of RDPE in the supernatant of strains 1A70SR and 1A75SR (Fig. 2b). These observations were consistent with the analysis of RDPE enzyme activity (Fig. 2c). Notably, although the biomass of the mutant strain 1A70SR increased, the secretion level was not substantially higher than in the strain 1A75SR, indicating that RDPE secretion is not considerably mediated by cell lysis. Additionally, the recombinant expression plasmid also contains the gene encoding Lac repressor LacI (38.6 kDa) from *E. coli*^33^. Even though the LacI protein was successfully expressed in the cells, no protein band for LacI was detected in the supernatant (Fig. 2b). To exclude the possibility that LacI was highly unstable extracellularly, we determined its stability in the late-stationary phase culture at 37 °C. The results show that the extracellularly added LacI was stable for at least 120 minutes (Fig. 2d), supporting the absence of LacI protein as a valid indicator for the lack of cell lysis. These results strongly suggest that the secretion of RDPE is not a result of unspecific cell lysis alone.

A recent report showed that α-enolase lacking a classical signal sequence from *Bacillus anthracis* can be detected in membrane vesicles (MVs)^34^. To exclude the possibility that RDPE secretion was also caused by vesicular transport systems, the MVs were isolated by ultracentrifugation. The distribution of protein RDPE in the culture supernatant and the isolated MVs was determined by SDS-PAGE analysis (Fig. 2e). Under these conditions, less than 10% of RDPE was detected in the MVs fraction. Thus, it is unlikely that RDPE is exported via MVs. As a consequence, the secretion of RDPE is not simply due to cell lysis but is mediated by an unidentified secretion pathway, i.e., a non-classical secretion pathway.

### RDPE homotetramerizes *in vitro* and *in vivo*

**Figure 3:**
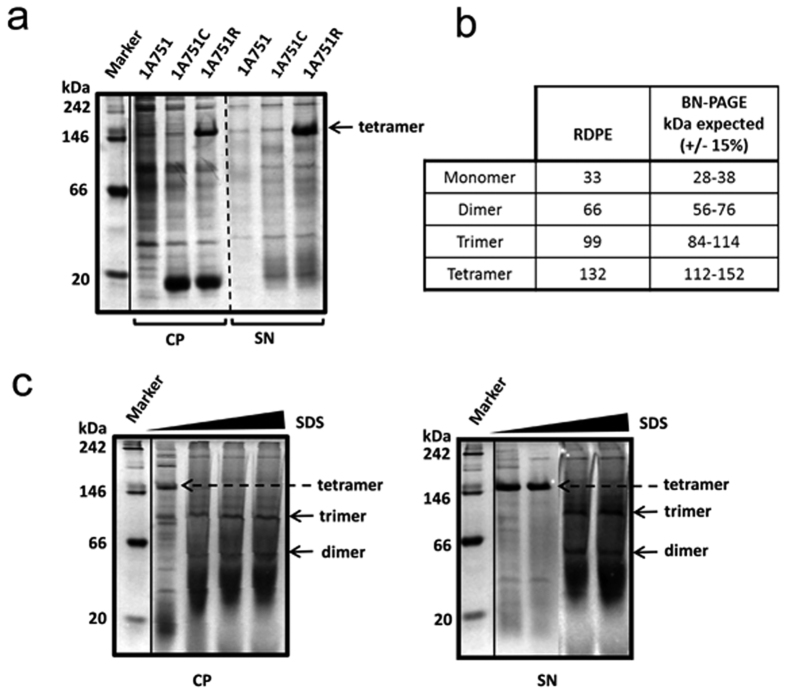
The oligomeric states of RDPE *in vivo* and *vitro.* (**a**) Analysis of soluble RDPE in the cytoplasmic fraction (CP) and culture supernatant (SN) with 24 h incubation by BN-PAGE. (**b**) Predicted masses of RDPE oligomeric forms and corresponding masses expected by BN-PAGE analysis. (**c**) The soluble protein fractions were combined with SDS concentrations of 0, 0.5%, 1% and 2% and run on a 10% acrylamide native gel. The tetramer, trimer and dimer bands of RDPE are labeled with arrows.

Structural studies of the DPEase family of enzymes revealed that some members of this protein family exist as homotetramers, as shown for *Agrobacterium tumefaciens* DPEase and *Clostridium cellulolyticum* DPEase^35^,^36^. To test whether RDPE expressed in *B. subtilis* can form such structures, we assessed the oligomeric state of RDPE using blue-native polyacrylamide gel electrophoresis (BN-PAGE). BN-PAGE can be used to determine the oligomeric states of protein complexes and allows for an estimation of the molecular mass of a complex ±15%^37^. The RDPE protein migrated as a single band at approximately 150 kDa (Fig. 3a), consistent with the expected tetramer mass (Fig. 3b). To confirm this result, we denatured the tetramer by adding SDS to the samples. As expected, the band at approximately 150 kDa was dissociated into bands of lower molecular mass with increasing SDS concentration. Two other oligomeric forms were observed for RDPE with masses of approximately 106 kDa and 60 kDa, consistent with the expected masses of trimer and dimer, respectively (Fig. 3b,c). In contrast, the monomeric RDPE form was not observed; this may be explained by rapid degradation due to structural instability (Fig. 3c). Together, these results demonstrate a strong preference for homotetrameric RDPE, and tetramerization is important for protein stability.

### C- and N-terminal residues of RDPE are crucial for its secretion

**Figure 4:**
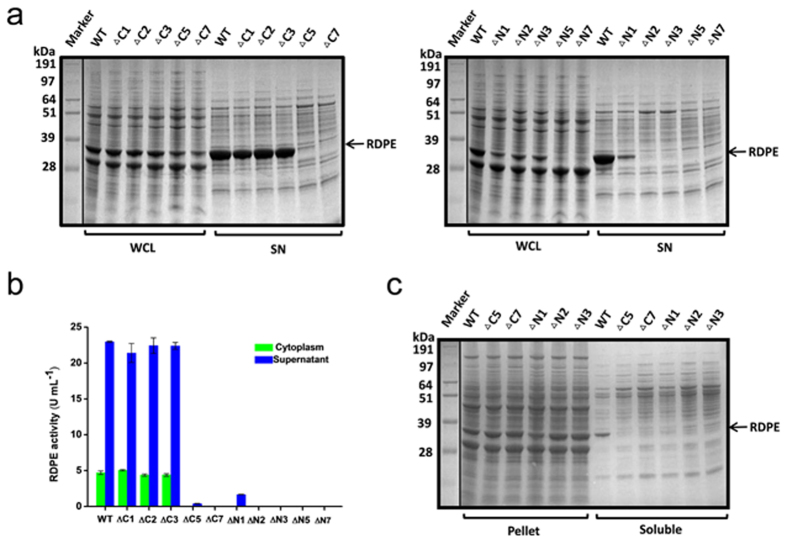
Effect of C- and N- terminal truncations of RDPE on its secretion. (**a**) SDS-PAGE analysis of the C- and N-terminal truncated RDPE proteins in the whole cell lysates (WCL) and culture supernatant (SN) fractions with 48 h incubation. (**b**) Enzyme activity of truncated RDPE in the cytoplasm and culture supernatant. Error bars are SDs from three independent experiments. (**c**) SDS-PAGE analysis of the pellet and soluble fractions from the cell lysates of dedicated strains expressing truncated mutants. All the samples were collected after 48 h of cultivation. WT is the strain expressing wild-type RDPE protein. The band of RDPE (33 kDa) is labeled with an arrow.

It is not known how folded RDPE is exported via a non-classical secretion pathway in *B. subtilis*. We wondered whether the C- and N-terminus of mature RDPE functioned as targeting sequences that are responsible for secretion. To test this, we probed a series of C- and N-terminal deletions in RDPE. We established that all tested variants are indeed expressed in *B. subtilis* except for the variants lacking five or seven N-terminal amino acids (Fig. 4a). Deletion of five amino acids from the C-terminus resulted in inhibition of extracellular secretion of the RDPE protein. In contrast, truncation of even a single amino acid from the N-terminus strongly affected the secretion of RDPE, indicating that the N-terminal region is likely required for RDPE translation or translocation. Furthermore, no RDPE variant missing seven residues at the C-terminus or two or more residues at the N-terminus was detected in the culture supernatant (Fig. 4a). Notably, accumulation of the RDPE variants in cells varied, which may reflect reduced stability of some truncations. Indeed, low accumulation of RDPE mutants disrupting secretion were detected in the cells (Fig. 4a). These results are consistent with data from the enzymatic assays (Fig. 4b), indicating that these mutants may have an impact on RDPE stability and are more susceptible to degradation. Additionally, truncation of five C-terminal amino acids or a single N-terminal amino acid resulted in loss of intracellular enzymatic activity. We could not detect RDPE activity in the cytoplasm or culture supernatant of RDPE variants with deletion of seven residues at the C-terminus or two or more residues at the N-terminus (Fig. 4b). We next examined the solubility of these truncated RDPE variants. However, no soluble fractions from the cell lysates of RDPE variants disrupting secretion were observed (Fig. 4c). These observations support the notion that the defect in secretion of these variants is likely due to protein stability issues or proteolysis. These results indicate that the C- and N- terminal sequences of RDPE are crucial for its secretion. The data also suggest that structural stability is likely to account for the differential cellular accumulation and secretion ability of these RDPE variants.

### Hydrophobic residues in the hydrophobic domains of RDPE is important for protein folding

**Figure 5:**
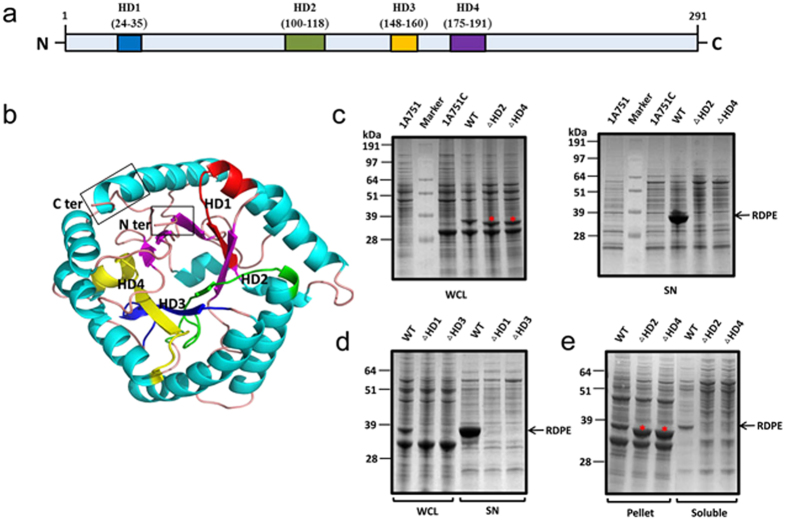
Two hydrophobic domains are required for structural stability of RDPE. (**a**) Domain structure of the RDPE protein. The four hydrophobic domains (HD1-HD4) are indicated. (**b**) Homology modeled monomeric structure of RDPE was based on the template of D-Psicose 3-epimerase from *Clostridium cellulolyticum* H10 (PDB ID: 3VNK) and *Agrobacterium tumefaciens* (PDB ID: 2HK1) using EasyModeller4.0. Helices are presented in green, sheets in magentas and loops in bright orange. N- and C-terminal domains are marked. The segments of HD2 and HD4 are displayed in green and yellow, respectively. (**c**) and (**d**) The whole cell lysates (WCL) and culture supernatant (SN) fractions from the *B. subtilis* 1A751 harboring the plasmids expressing either the full length or the deleted RDPE were examined by SDS-PAGE after 48 h of growth. (**e**) SDS-PAGE analysis of the pellet and soluble fractions from the cell lysates of strains *B. subtilis* 1A751 harboring the plasmids expressing the RDPE variants with deleted hydrophobic domains HD2 or HD4. The band of RDPE (33 kDa) is labeled with an arrow. (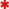) The deleted RDPE in a slightly smaller size is indicated.

Previous studies of the non-classical protein enolase of *B. subtilis* have shown that an internal hydrophobic helical domain is essential for efficient secretion. We hypothesized that hydrophobic domains may contribute to the secretion of RDPE, and we thus performed an *in silico* analysis of the RDPE sequence (291 amino acids). The hydrophobicity analysis performed by ProtScale (http://web.expasy.org/protscale/) predicted that RDPE has at least six extremely hydrophobic regions (Supplementary Fig. S2a), which are presumed to be transmembrane segments. Next, several programs (HMMTOP^38^, TMpred^39^, TMHMM^40^ and SPLIT^41^) were used to predict the transmembrane segments. However, the four programs considered the presence of transmembrane segments to be uncertain. We found that only four hydrophobic segments were predicted by the SPLIT program (Fig. 5a, Supplementary Fig. S2b). Furthermore, the hydrophobic domains are nearly conserved in the DPEase family, whereas the length of the polypeptide varies (Supplementary Fig. S2c). Using the crystal structure of D-Psicose 3-epimerase from *Clostridium cellulolyticum* H10 as a template, a predicted three-dimensional (3D) monomeric structure was generated using EasyModeller4.0^42^. The modelling revealed that these hydrophobic domains primarily are localized to the loop and sheet regions, but not the helical domains of RDPE protein (Fig. 5b). To investigate the physiological role of these hydrophobic segments (Fig. 5a), we examined the influence of each domain on the secretion of RDPE. Plasmids containing the *rdpe* gene in which the HD1, HD2, HD3 or HD4 had been deleted were used for expression of the mutated RDPE in *B. subtilis*. The mutated proteins ΔHD2 and ΔHD4 were identified by their slightly smaller size compared to the full-length RDPE. The results showed that deletion of the two hydrophobic segments completely abolished the secretion of mutant RDPE, while the wild-type RDPE was secreted into the medium under identical conditions (Fig. 5c). However, we could not detect the mutant RDPE deletion of HD1 or HD3 in the cells or the culture supernatant (Fig. 5d), which may be caused by rapid degradation due to major structural impairment of the remaining protein. These results raised the possibility that the RDPE variants ΔHD2 and ΔHD4 undergo significant conformational changes. Hence, we determined the solubility of the RDPE variants ΔHD2 and ΔHD4, which were both insoluble in the cell (Fig. 5e). Deletion of these hydrophobic domains from RDPE suppressed both its secretion and folding.

**Figure 6:**
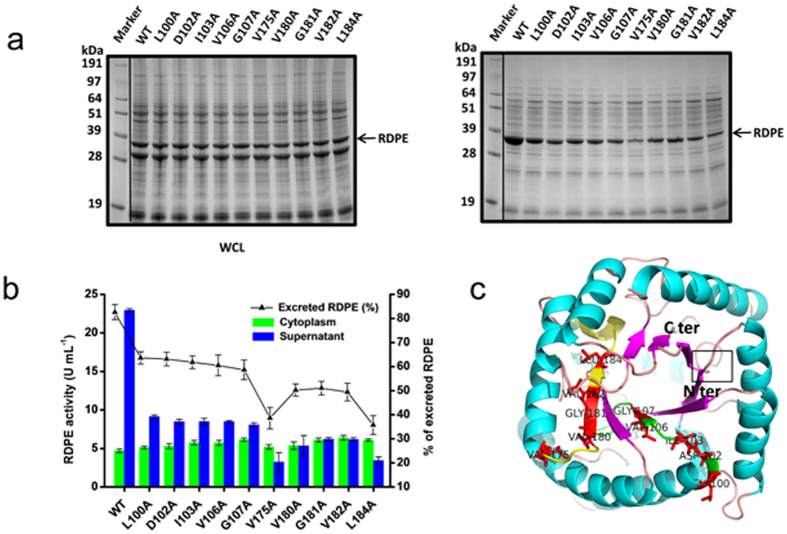
Effect of hydrophobic amino acids in the hydrophobic domains on the secretion of RDPE. (**a**) Several variants disrupt the secretion of RDPE. The whole cell lysates (WCL) and culture supernatant (SN) fractions of indicated strains expressing variants with single alanine-substitution were analysed by SDS-PAGE after 48 h of growth. Most of these residues are hydrophobic. The band of RDPE (33 kDa) is labeled with an arrow. (**b**) The relative amount of wide-type RDPE and alanine-substituted variants was followed by enzyme activity analysis. Error bars are SDs from three independent experiments. (**c**) Predicted tertiary structure of monomer RDPE. The structure was generated as described for the panel Fig. 5b. Hydrophobic residues that can suppress the efficiency of secretion are shown using the stick molecular representation.

**Figure 7:**
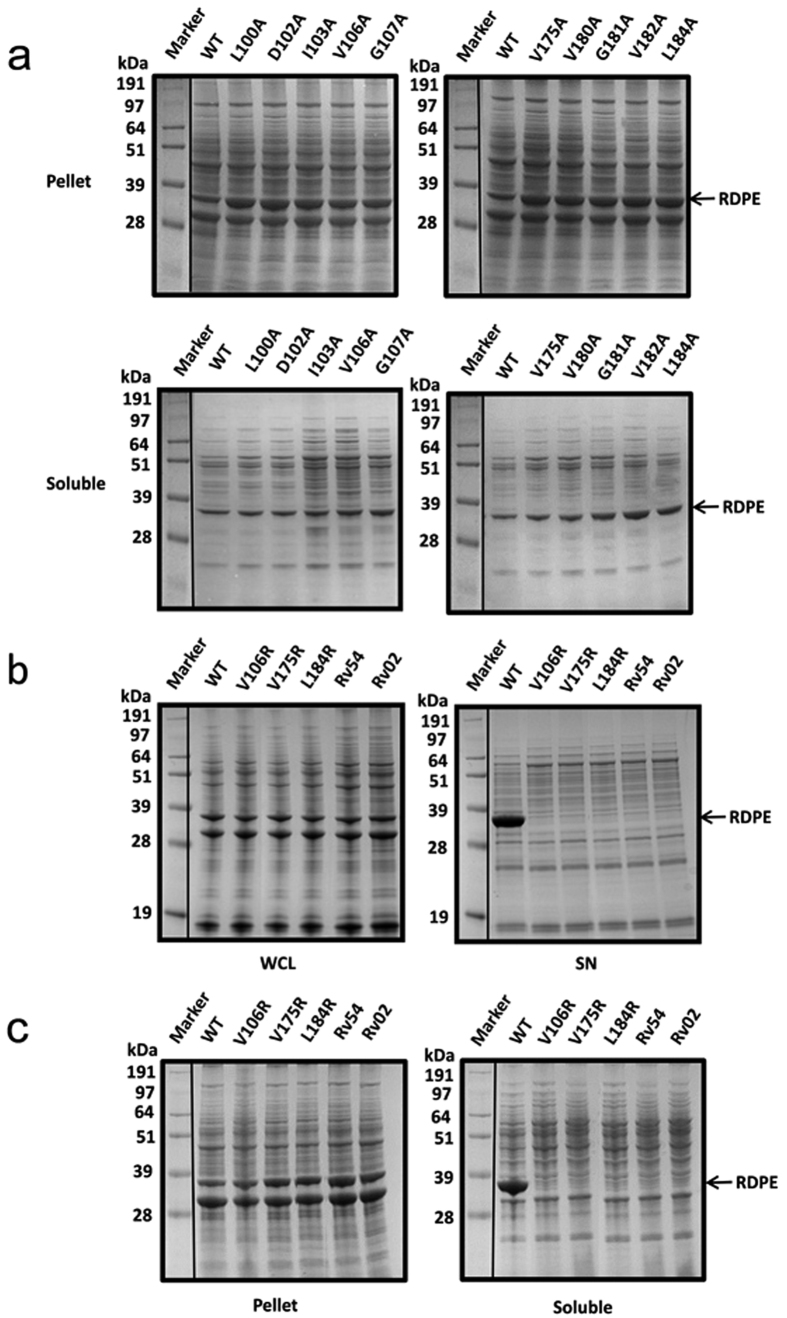
The hydrophobic amino acids change the dynamic balance between the folded and unfolded state of RDPE. (**a**) SDS-PAGE analysis of the pellet and soluble fractions from the cell lysates of the indicated strains expressing secretion-disrupting variants. (**b**) Expression of arginine-substituted mutations (V106R, V175R and L184R) and doubly or triply alanine-substituted variants (Rv54 or Rv02) in *B. subtilis* 1A751. (**c**) The pellet and soluble fractions from the cell lysates of the *B. subtilis* 1A751 harboring the plasmids expressing V106R, V175R, L184R, Rv54 and Rv02 mutations were analysed by SDS-PAGE. All the samples were collected after 48 h of cultivation. WT is the strain expressing wild-type RDPE protein. The band of RDPE (33 kDa) is labeled with an arrow.

To better evaluate the role of individual residues, we prepared a set of alanine scanning mutants covering the two hydrophobic domains HD2 and HD4. All individual alanine variants were successfully expressed in *B. subtilis*. No difference in size or expression level was observed between wild-type and mutant RDPE (Supplementary Fig. S3). Substitution of alanine for residues Leu^100^, Asp^102^, Ile^103^, Val^106^ and Gly^107^ had significant effects on secretion; secretion efficiency declined to approximately 60% from 83% for wild-type RDPE. The secretion efficiency was reduced further to approximately 50% with substitution of alanine for residues Val^180^, Gly^181^ and Val^182^. An even greater effect on secretion was observed for variants V175A and L184A; the secretion efficiency fell to less than 40% (Fig. 6a,b). These residues are located away from the monomer surface (Fig. 6c), and we predicted, that they did not affect the ability of RDPE to assemble correctly. However, this class of mutants (100A, D102A, I103A, V106A, G107A, V175A, V180A, G181A, V182A and L184A) showed extensive accumulation in the cells (Fig. 7a), indicating that these residues are responsible for the stability of RDPE. Thus, we hypothesized that protein stability issues are likely to account for the low secretion efficiency. To assess the stability of these variants, we chose to study soluble crude extracts, which were prepared and added to the late-stationary phase (48 h) culture at 37 °C. The alanine-substituted mutants and wild type were stable for at least 2 h without apparent degradation (Supplementary Fig. S4), indicating that these mutations did not considerably affect the high stability of the soluble RDPE *in vitro*. Therefore, it appears that these RDPE mutations do not strongly change their conformation but do change the dynamic balance between its folded and unfolded states. Moreover, except for Asp^102^, all of these residues are hydrophobic amino acids, demonstrating that hydrophobic amino acids in these segments play an important role in RDPE folding. To confirm this result, we constructed V106R, V175R and L184R mutants in which the residues were replaced by the polar residue arginine and Rv54 (V175A/L184A) and Rv02 (V180A/G181A/V182A) in which the residues were doubly or triply substituted by alanine residues. As expected, switching the hydrophobic residues to the polar residue arginine at the Val^106^, Val^175^ and Leu^184^ positions abolished the secretion of RDPE (Fig. 7b). Notably, the additive effects of double and triple substitutions on RDPE secretion were observed, blocking the secretion (Fig. 7b). We could not detect any soluble RDPE in the crude extracts (Fig. 7c), indicating that the assembly of RDPE relies on these hydrophobic amino acids. Collectively, our results suggest that the presence of hydrophobic residues in the hydrophobic segments is essential for RDPE folding.

### Tetramerization of RDPE is critical for its non-classical secretion

**Figure 8:**
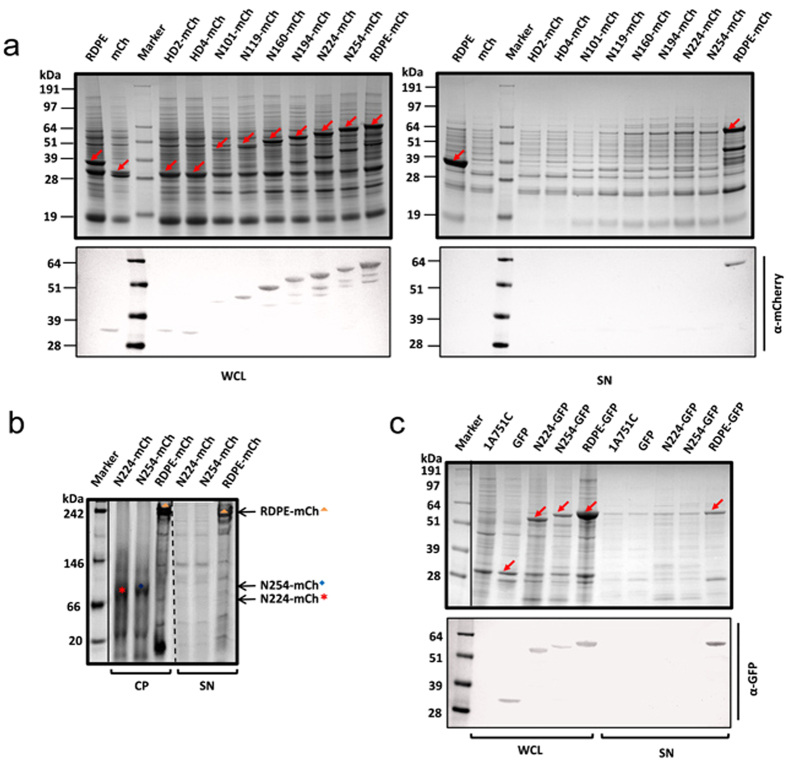
Folded RDPE is required for directing reporter proteins translocate across the cytoplasmic membrane. (**a**) Two hydrophobic HD2 and HD4 domains, full-length RDPE and different lengths of N-terminus of RDPE were fused to mCherry. Recombinant mCherrys were overexpressed in *B. subtilis* 1A751. The whole cell lysates (WCL) and culture supernatant (SN) fractions with 48 h incubation were separated by SDS-PAGE and western blot. Corresponding bands are labeled with arrows. (**b**) The fusion proteins in the cytoplasmic fraction (CP) and culture supernatant (SN) with 24 h incubation were analysed by BN-PAGE. The RDPE-mCh tetramer band (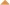), N224-mCh monomer band (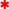) and N254-mCh band (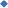) are indicated. (**c**) Expression of GFP fused proteins in *B. subtilis* 1A751. The N-terminus of RDPE (N224 and N254) and the full-length RDPE were fused to GFP. The samples were collected after 48 h of cultivation. 1A751C strain harboring empty vector pMA5 is regarded as a negative control. Corresponding bands are labeled with arrows.

Based on the result that two hydrophobic segments are important for RDPE folding, we hypothesized that the two segments HD2 and HD4 may play a vital role in RDPE excretion. To test this hypothesis, a plasmid pMA5mCh harboring the reporter gene *mCherry* was constructed. The two hydrophobic segments HD2 and HD4 and the full-length RDPE were fused onto the mCherry protein to confirm whether these sequences can be used as signals to direct release of mCherry into the culture medium (Supplementary Fig. S5a). Indeed, all of the recombinant proteins were successfully expressed in *B. subtilis* (Fig. 8a). We found that although mCherry itself cannot be secreted into the medium, the full-length RDPE sequence could direct mCherry into the growth medium (Fig. 8a). However, we found that neither HD2 nor HD4 promoted mCherry export across the *B. subtilis* membrane (Fig. 8a), suggesting that these hydrophobic segments were insufficient to direct mCherry secretion. We dissected the secondary structure of RDPE to determine the minimal length for mCherry export and next constructed six additional hybrid proteins with different lengths of RDPE N-terminus (N101-mCh, N119-mCh, N160-mCh, N194-mCh, N224-mCh and N254-mCh) (Supplementary Fig. S5a). All fusion proteins were analysed by SDS-PAGE and western blot. Notably, none of the constructs could direct mCherry across the cytoplasmic membrane, although they were all successfully expressed in *B. subtilis* (Fig. 8a). Analysis of the relative fluorescence units (RFU) of these proteins showed that the fusion proteins still retained the biological activity of mCherry (Supplementary Fig. S5b). However, minor bands of lower apparent molecular weight that likely corresponded to degradation products, were also visible in the cells, as revealed by immunoblot analysis (Fig. 8a). This suggests that the fusion proteins are unstable in the cytoplasm, raising the possibility that the released proteins were degraded by extracellular proteases. Moreover, the oligomeric state of these recombined mCherrys was assessed by BN-PAGE. The fusion protein RDPE-mCh existed as a stable tetramer in the cytoplasm and culture supernatant. In contrast, only the recombinant proteins N224-mCh and N254-mCh were detected in the cytoplasm in a monomeric state (Fig. 8b). All other fusions were unstable and were not detected in the cell under nondenaturing conditions (Supplementary Fig. S5c). We observed that the full-length RDPE sequence also directed a reporter green fluorescent protein (GFP) to the culture medium. In contrast, the constructs with N-terminal 224 residues (N224-GFP) and 254 residues (N254-GFP) could not serve as signals for directing the secretion of GFP (Fig. 8c), although the fusion proteins still displayed the biological function of GFP (Supplementary Fig. S5d). The degradation products of GFP fusion proteins were not found (Fig. 8c), indicating that these recombinant proteins possess high extracellular stability. Thus, the defect in secretion of these fusion proteins is probably not due to proteolysis. These data strongly suggest that an intact RDPE sequence is required for directing protein secretion and that folding of RDPE plays a vital role in its secretion.

**Figure 9:**
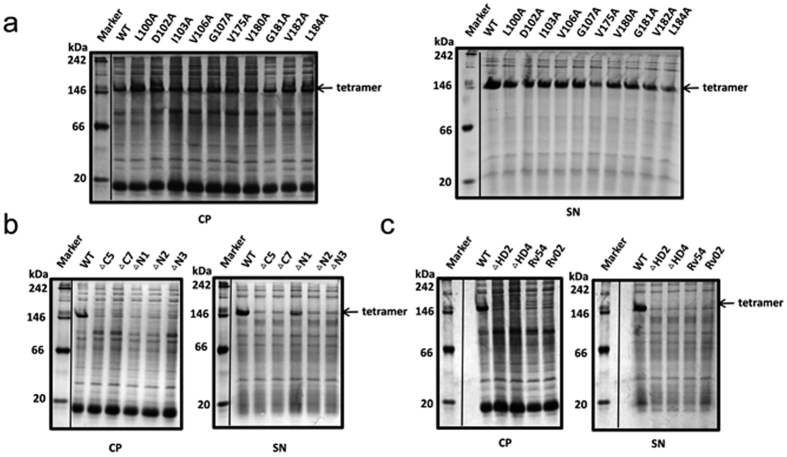
Tetramerization of RDPE is important for its non-classical secretion. The soluble RDPE in the cytoplasmic fraction (CP) and culture supernatant (SN) of strains expressing the single alanine-substituted secretion-disrupting mutants (**a**), the truncated mutants (**b**) and the deletion and doubly or triply alanine-substituted mutants (**c**) were analysed by BN-PAGE after 24 h of cultivation. The band of tetramer is labeled with an arrow.

Given that RDPE homotetramerizes *in vitro* and *in vivo* and that folded tetramer RDPE could successfully direct mCherry to the medium, we hypothesized that the inability of RDPE variants to tetramerize may result in the lack of secretion of these mutant proteins. Therefore, we wondered whether tetramerization of the RDPE mutants was impaired. To this end, cellular and secreted fractions of the strains expressing RDPE variants that disrupt secretion (the alanine-substituted mutants 100A, D102A, I103A, V106A, G107A, V175A, V180A, G181A, V182A, L184A, Rv54 and Rv02; the truncation/deletion mutants ΔC5, ΔC7, ΔN1, ΔN2, ΔN3, ΔHD2 and ΔHD4) were analysed under nondenaturing conditions. The soluble fractions in the cytoplasm were further analysed by BN-PAGE. Strikingly, each of the single alanine-substituted derivatives of RDPE retained the ability to tetramerize. These RDPE variants all formed homotetramers, as did wild-type RDPE (Fig. 9a). As for the truncation mutants, we only observed a weak band corresponding to the tetrameric form of RDPE in the supernatant of the ΔN1 mutant (Fig. 9b). As a control, we have confirmed that the secretion-blocking mutations (ΔHD2, ΔHD4, Rv54 and Rv02) lost the ability to form tetramers (Fig. 9c). These data suggest that the formation of tetrameric structure contributed to the stability of RDPE. Taken together, the results indicate that non-classical RDPE secretion does require protein folding, supporting the notion that tetramerization is a prerequisite requirement for RDPE secretion.

### Secretion of an irreversibly cross-linked RDPE tetramer

**Figure 10:**
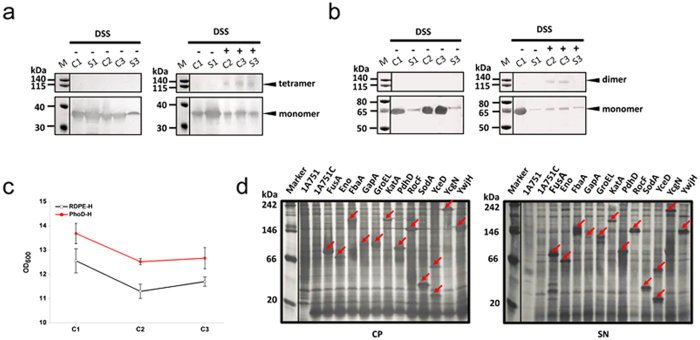
Multimeric substrate secretion by non-classical secretion pathway. (**a**) Irreversibly cross-linked RDPE tetramer could be secreted. Immunoblotting analysis of the control whole cell lysates (C1) and culture supernatant (S1) samples, DSS cross-linked cells (C2), cells recovered in the absence of DSS (C3), and secreted RDPE in the supernatant (S3). The tetrameric RDPE was finally found in the culture supernatant (S3). (**b**) As a negative control, the cross-linked dimeric PhoD protein was not accumulated in the medium (S3). The indicated fractions were prepared as described for panel a. (**c**) Growth of *B. subtilis* strains expressing proteins RDPE-H and PhoD-H. The strains were treated with crosslinker DSS. At indicated time, the cell samples were collected for optical measurement. Error bars are SDs from three independent experiments. (**d**) BN-PAGE analysis of homologous non-classically secreted proteins in *B. subtilis* after 24 h of cultivation. The parent strain 1A751 and 1A751C harboring empty vector pMA5 are regarded as negative controls. The corresponding bands of FbaA (pentamer), GapA (trimer), GroEL (dimer), KatA (tetramer), PdhD (dimer), RocF (pentamer), SodA (dimer), YceD (dimer and monomer), YcgN (tetramer) and YwjH (heptamer) are labeled with arrows.

Based on the result that RDPE secretion is dependent on an intact multimeric complex, we hypothesized that the fully folded tetrameric proteins would be maintained through the non-classical secretion pathway after recognition. Therefore, we tested whether RDPE homotetramers that are irreversibly cross-linked inside the *B. subtilis* cells would still be exported by the non-classical export pathway. We examined the cytoplasmic and secretion fractions by western blot analysis, using anti-His antibody as a probe (the anti-RDPE antibody is difficult to obtain). Thus, we created a RDPE derivative with a C-terminal 6-His tag (RDPE-H), which is as active as wild-type RDPE. We next expressed the recombinant RDPE-H under the control of the promoter *P*_*HpaII*_, and cross-linked the RDPE tetramer by introducing the cross-linking agent disuccinimidyl substrate (DSS) *in vivo*. After removing excess crosslinker and the extracellular RDPE protein from the cells, the cells were added to the fresh medium without the crosslinker. Subsequently, we analysed the oligomeric state of proteins in the cytoplasmic and secretion fractions at each stage of this experimental process to determine whether cross-linked RDPE could be secreted into the medium. As expected, a band was seen with a molecular weight corresponding to the size of homotetramer RDPE in the medium fraction and this homotetramer could not be reduced by β-mercaptoethanol (Fig. 10a), indicating that the crosslinker DSS indeed irreversibly cross-linked four RDPE subunits and cannot be reversed by excess reducing agent. Unfortunately, the crosslinker DSS was toxic and inhibited cell growth, which resulted in cell lysis (Fig. 10c). Therefore, the possibility that secretion of irreversibly cross-linked protein is due to cell autolysis could not be ruled out, although growth gradually increased after inoculation into the fresh medium (Fig. 10c). To determine whether cell lysis plays an important role in the secretion of cross-linked RDPE, we used a strain expressing an endogenous protein alkaline phosphatase D (PhoD) with a His tag as control. The phosphodiesterase PhoD has been identified as a TatAdCd-dependent substrate in *B. subtilis*^3^. In this case, the two strains showed a similar growth pattern (Fig. 10c). The cross-linked dimer PhoD was found in the C2 and C3 fractions after treatment with DSS but did not accumulate in the medium (Fig. 10b). It is now clear that the excretion of cross-linked substrate is likely not a result of cell lysis. Collectively, these results demonstrate that RDPE can be excreted as a tetramer unit by a non-classical secretion system.

### Secretion of multimeric homologous non-classically secreted proteins

Based on the result that the heterologous non-classically excreted protein RDPE was tetrameric and exported as a single unit, we hypothesized that the homologous non-classical proteins in *B. subtilis* would also be exported in their multimeric state. In *B. subtilis*, 17 typical cytoplasmic proteins containing no classical signal peptide were detected in the extracellular medium via secretome analysis^19^ and are usually regarded as non-classically secreted proteins. To verify the excretion pattern of these proteins, we attempted to overexpress 15 cytoplasmic proteins: secondary transporter of divalent metal ions/citrate complexes (CitH), elongation factor G (FusA), enolase (Eno), fructose 1,6-bisphosphate aldolase (FbaA), glyceraldehyde 3-phosphate dehydrogenase (GapA), molecular chaperone (GroEL), vegetative catalase (KatA), pyruvate dehydrogenases (PdhA, PdhB and PdhD), arginase (RocF), superoxide dismutase (SodA), general stress protein (YceD), 1-pyrroline-5-carboxylate dehydrogenase (YcgN) and transaldolase (YwjH). The result indicated that CitH and PdhB were not successfully expressed in *B. subtilis*. Although PdhA was expressed in the cells, it could not be detected in the culture supernatant. The remaining 12 cytoplasmic proteins were all successfully expressed and secreted into the medium without fusion to any signal peptide (Supplementary Fig. S6), which illustrated that the 12 proteins indeed are non-classically secreted proteins in *B. subtilis*. Subsequently, we conducted BN-PAGE analysis of the 12 non-classically secreted proteins. The expected oligomeric masses of the homologous non-classical proteins by BN-PAGE are shown in Supplementary Fig. S7. Our experiments showed that FbaA (pentamer), GapA (trimer), GroEL (dimer), KatA (tetramer), PdhD (dimer), RocF (pentamer), SodA (dimer), YcgN (tetramer) and YwjH (heptamer) multimerized *in vitro* and *in vivo* (Fig. 10d). However, the YceD protein existed in monomeric and dimeric forms, and the proteins FusA and Eno maintained a monomeric state (Fig. 10d). Taken together, these results demonstrate a strong preference for non-classical secretion proteins to exist in a multimeric state.

### Subcellular localization of non-classically secreted proteins

**Figure 11:**
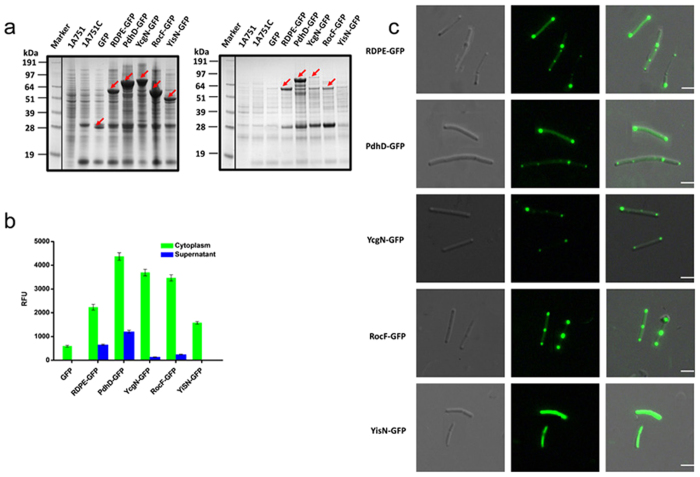
Cellular localization of non-classically secreted proteins with GFP fusion. (**a**) SDS-PAGE analysis of the GFP fused proteins in the whole cell lysates (WCL) and culture supernatant (SN) fractions with 48 h incubation. The parent strain 1A751 and 1A751C harboring empty vector pMA5 are regarded as negative controls. Corresponding bands are labeled with arrows. (**b**) Fluorescence measurement of fusion GFP proteins in the cells and culture supernatant. Cells were resuspended with equal volume of PBS (pH 8.0) buffer. The extinction and emission wavelength were set at 488 and 520 nm, respectively. Error bars are SDs from three independent experiments. (**c**) To detect the fluorescence signals, the cells were chosen randomly. All the non-classically secreted fusion proteins showed a considerable accumulation of fluorescence. Scale bar, 25 μm. DIC, Differential Interference Contrast.

It is unknown where non-classically secreted proteins are translocated. The green fluorescent protein (GFP) is a standard imaging tool for studying protein localization in living cells^43^, here, we performed microscopic imaging of protein fusions to GFP to study the localization of non-classical proteins. To this end, we fused *gfp* to the C-terminus of several non-classically secreted proteins: RDPE, PdhD, YcgN and RocF. Notably, all of the GFP-tagged proteins could be transported through the cell membrane (Fig. 11a). These GFP fusions retained the biological GFP function (Fig. 11b). In addition, we have shown that the RDPE fusion RDPE-GFP had RDPE enzyme activity^29^. Thus, the fusion technique does not likely influence the function of proteins. For the non-classically secreted proteins, GFP fluorescence was distributed at the cell poles and the division septum (Fig. 11c). The accumulation at the poles and septum site indicates that the non-classical proteins were preferentially exported at the poles and septum region. As a control, the non-secreted cytoplasmic protein YisN did not preferentially accumulate at the poles and division septum but rather was dispersed in the cytoplasm (Fig. 11c). These data demonstrate that non-classically secreted proteins have similar subcellular localization and appear to translocate to the poles and septum site.

## Discussion

The excretion of non-classically secreted proteins is a long-known and common phenomenon in bacteria. In recent years, there have been an increasing number of studies of non-classical secretion^9^,^28^. However, the crucial questions remain: How are these non-classical proteins excreted? Are they released simply due to cell lysis? What is the selection principle for the non-classical secretion pathway? Using RDPE as a model protein, this work reveals that non-classical protein secretion is the result of an unknown active system and not simply due to cell lysis. Here, we provide evidence for an original multimer substrate recognition mechanism for the non-classical secretion system in *B. subtilis*. We show that the non-classical secretion pathway can export an intact tetramer protein complex and that the multimeric substrate can be recognized by the secretion machinery, suggesting that this novel multimeric selection principle may serve as a generally applicable mechanism for non-classical protein secretion.

Secretome studies showed that a number of typical cytoplasmic proteins lacking a signal sequence were found in the culture medium of bacteria^25^. Because the non-classically secreted proteins do not possess a common signal, the general opinion of their secretion is most likely due to cell lysis. However, an increasing preponderance of evidence contradicts the excretion that is simply mediated by cell lysis^20^,^28^,^44^,^45^. We now show that RDPE was found to be substantially secreted by *B. subtilis* without a typical signal peptide. Importantly, several lines of evidence demonstrate that high extracellular levels of RDPE are not simply due to cell lysis. Instead, RDPE is actively exported by a non-classical secretion pathway, consistent with previous observation of the secretion of carboxylesterase Est55 in *B. subtilis*^24^. We observed that the cell density gradually declined in the late-stationary phase, but the secretion efficiency of RDPE was still maintained at a stable level and did not dramatically increase (Fig. 1b,c), suggesting that cell lysis alone does not account for protein secretion. Second, the *lytC lytD* double mutant strain showed a slowing rate of lysis but a similar secretion pattern compared with that of the parent strain (Fig. 2a,b). Under these conditions, we show that the intracellular protein LacI with high extracellular stability could not be detected in the medium, indicating that autolysis is not a major factor. Third, the secretion-disrupting variants with single alanine-substitution exhibited differential secretion levels that provided evidence that RDPE is excreted through a specialized pathway. Finally, the non-classically secreted proteins accumulated at the poles and the septum region (Fig. 11c), suggesting that cell lysis is too simple an explanation for their secretion. We propose that these proteins are excreted via an active secretion mechanism.

Early studies of non-classically secreted proteins suggested that the N-terminal sequences were responsible for transport through the cytoplasmic membrane^24^,^46^. However, distinct “non-classical signal peptides” show little similarity in amino acid sequence or characteristic features. We speculate that different non-classical secretion pathways diverge considerably, and individual mechanisms may possess unique signals for secretion. The recognition signals for substrate excretion have not yet been identified in *B. subtilis*. The strong secretion of RDPE in *B. subtilis* inspired us to study its structural properties for secretion, to explain the mystery of the non-classical proteins. We found that the C- and N-terminal sequences are required for efficient secretion of RDPE. Strikingly, truncation of five amino acids from the C terminus or a single amino acid from the N terminus of RDPE disturbs its secretion (Fig. 4a). However, neither the C-terminal nor N-terminal region could guide the reporter protein GFP across the cytoplasmic membrane as a signal peptide (Supplementary Fig. S8). This distinguishes RDPE from a non-classical protein cellulase (Cel-CD) that has an N20 peptide that can serve as a signal peptide for the secretion of target proteins^47^. Unlike *B. subtilis* enolase (EnoBs), which has a hydrophobic membrane-embedded domain^48^, sequence analysis of RDPE showed that it possesses hydrophobic domains. We found that two segments, namely HD2 and HD4 are crucial for the folding of RDPE. Moreover, alanine scanning analysis of the two segments showed that the hydrophobic amino acids play an essential role in the stability of RDPE (Fig. 7a). It appears that the amino acid composition of the hydrophobic domains of RDPE alters the equilibrium between its folded and unfolded state. Because the crystal structure of RDPE has not yet been identified, a predicted tertiary structure of homotetramer RDPE was generated based on the template of D-Psicose 3-epimerase from *Clostridium cellulolyticum* H10 (PDB ID: 3VNK) by the HOMCOS server^49^. The Try-118 (in the HD2 domain) and Ile-191(in the HD4 domain) residues from each of the two RDPE dimers (A-B, C-D) were found in close proximity (Supplementary Fig. S9), supporting the notion that the two hydrophobic domains might be involved in a dimeric interface between two subunits in a dimer.

Exported proteins of *B. subtilis* are potential substrates of extracytoplasmic proteases. These secreted proteases can degrade misfolded proteins released into the culture medium^50^. Here, we found that folded RDPE has high extracellular stability (Supplementary Fig. S1). The secretion-blocking mutations were aggregated in its insoluble state (Figs 4c,5e and 7c), indicating that these mutations indeed significantly perturb its structural stability. However, no degradation products were detected in the supernatant (Figs 4a,5c and 7b), supporting the notion that proteolysis may not be the principal factor for disrupting secretion. Thus, the structural stability of proteins may have substantial importance for the export of RDPE.

Previous studies have indicated that RDPE can direct various proteins into the culture medium from *B. subtilis* via the non-classical secretion pathway^29^. However, the minimal length of RDPE for carrying recombinant proteins to export is not yet clear. Our experiments show that only intact full-length RDPE could direct the export of cytoplasmic proteins mCherry and GFP into the medium (Fig. 8a,c). This is different from the proteins Cel-CD and EnoBs. Interestingly, the fusion protein RDPE-mCh tetramerized both in the cytoplasm and culture supernatant (Fig. 8b). This is, to our knowledge, the first report of tetrameric fusion protein secreted into the medium by *B. subtilis*. Our extensive analysis of secretion-disrupting or secretion-blocking mutations confirmed that tetramerization of RDPE is required for its non-classical secretion, indicating that RDPE is similar to the YkuE substrate of the ESX system in *B. subtilis*. The ESX system can export an intact YkuE dimer^51^, and the *yuk/yue* locus (*yukD, yukC, yukBA, yueB, yueC* and *yueD*) encodes functional components of an ESX secretion system in *B. subtilis*^52^. The possible role of an ESX system in RDPE secretion was examined in each of the *yuk/yue* knockout strains. We found that none of these mutations impaired the translocation rate (Supplementary Fig. S10), indicating that the ESX system is not involved in the secretion of this class of non-classical proteins. Furthermore, we verified that the secretion of RDPE is independent on Sec or Tat pathway^29^. Taken together, the results, combined with other studies, indicate that tetramerization is a prerequisite for RDPE secretion by a non-classical secretion system and is responsible for recognition and secretion of RDPE as well.

In bacteria, the vast majority of secretory proteins are exported either via the Sec-pathway or the Tat-pathway. The Sec system translocates unfolded proteins across the cytoplasmic membrane, while the Tat system exports fully folded proteins^4^. In the case of non-classical secretion pathways, no clear substrate specificities have yet been identified. Our results suggest that RDPE homotetramerized *in vitro* and *in vivo* (Fig. 3a), and we hypothesized that the non-classical secretion pathway may export tetramers. We used the crosslinker DSS to cross-link RDPE, and the tetramerization was captured *in vitro* and *in vivo*. If the non-classical secretion pathway can secrete irreversibly cross-linked RDPE tetramers, this suggests that the components of the non-classical secretion pathways could export an intact tetramer. To our knowledge, this is the first evidence that a non-classical secretion pathway can excrete a folded tetramer protein.

To characterize the pattern of the non-classical secretion pathway, we analysed the oligomeric state of homologous non-classically secreted proteins in *B. subtilis*. Most of the proteins existed as a multimer in the cytoplasm and supernatant (Fig. 10d), signifying that the non-classical secretion system is responsible for the secretion of multimeric substrate proteins. Thus, as far as we know, this is the first time to describe the excretion of typical cytoplasmic proteins in a multimeric state in *B. subtilis*. Strikingly, the non-classical secretion pathway also gained the ability to export monomeric proteins (FusA, Eno and YceD). One possibility is that this secretion pathway has broader substrate specificity, similar to the Tat-pathway, permitting the exportation of incorrect substrate^53^. In bacteria, the non-classical protein export system has a variety of secretory protein substrates. However, little is known about the selection principles that might be involved in non-classical secretion^28^. Our results suggest that multimerization of proteins would be an important characteristic for a non-classical secretion system, and that these non-classically secreted proteins may share a similar pattern of substrate recognition and secretion.

Non-classical protein secretion in bacteria, also referred to as “excretion of cytoplasmic proteins”, raises an intriguing question: what is the benefit of their excretion? This question has been addressed by extensive past work. It would waste energy and resources unless these secreted proteins play an important role in the extracellular environment^28^. In pathogenic bacteria, cytosolic proteins such as metabolic enzymes and molecular chaperones are moonlighting proteins and play a role in bacterial virulence^21^. Meanwhile, excretion of cytoplasmic proteins doubtless contributes to bacterial virulence. In *S. aureus*, two excreted cytoplasmic enzymes, FbaA and GAPDH, are involved in pathogenicity^54^. Enolase in *Streptococcus pneumoniae* binds to the human complement inhibitor C4b-binding protein and contributes to complement evasion^55^. The present study showed that various cytoplasmic proteins, such as the metabolic enzymes GapA, Eno, FbaA and PdhD and chaperone GroEL, are found in the extracellular environment (Supplementary Fig. S6), consistent with previous studies^16^,^17^,^18^. Nevertheless, the benefit of non-classical protein export in the non-pathogenic bacteria *B. subtilis* has not yet been identified; this subject remains to be explored.

In conclusion, our results demonstrate that non-classical secretion of RDPE is not simply due to cell lysis in *B. subtilis*. We assume that a selection principle that determines the secretion of non-classically secreted proteins must exist, as clearly folded tetramer RDPE can be recognized and excreted by a non-classical secretion pathway. Furthermore, various cytoplasmic proteins excreted by this system exist in a similar multimeric state. We conclude that the non-classical secretion pathway may prefer to export fully folded proteins in *B. subtilis*. The proposed model of recognition for the folded multimeric proteins can provide an explanation of the selection procedure in the non-classical secretion system.

## Methods

### Bacterial strains and growth conditions

All strains and plasmids used in this study are listed in Supplementary Table S1. *Escherichia coli* DH5α was used for DNA manipulation. *Bacillus subtilis* 1A751 was used as a host for expression. *E. coli* DH5α was grown on Luria-Bertani (LB) liquid medium (1% tryptone, 0.5% yeast extracts, and 0.5% NaCl) or on LB agar plates. *B. subtilis* strains were cultivated in a rotary shaker at 37 °C in SR medium consisting of 1.5% tryptone, 2.5% yeast extract and 0.3% K_2_HPO_4_, pH = 7.2. Plasmids were transformed into *B. subtilis* strains as previously described^56^. When appropriate, media for *E. coli* DH5α was supplemented with 100 μg ml^−1^ ampicillin; media for *B. subtilis* strains was supplemented with 50 μg ml^−1^ kanamycin, 12.5 μg ml^−1^ chloramphenicol, or 400 μg ml^−1^ spectinomycin. When required, 1 mM isopropyl-β-D-thiogalactopyranoside (IPTG) was added to induce RDPE expression.

### Plasmid and strain constructs

All cloning techniques and transformation of *E. coli* were performed as previously described^57^. The recombinant plasmids were generated using a general restriction enzyme-free and ligase-free method^58^. The pMAR plasmid, a derivative of the *E. coli/B. subtilis* shuttle vector pMA5 carrying a constitutive promoter *P*_*HpaII*_^29^, was used for gene manipulation. Oligonucleotide pairs are listed in Supplementary Table S2. The pMASR plasmid, which was used for inducible gene expression, was generated by ligation of an insertion fragment *P*_*spac*_-*lacI* cassette obtained by PCR from the plasmid pHCMC05 using primers MA121 and MA122 and the linear vector backbone of pMAR, amplified with primers MA123 and MA124. For construction of the RDPE mutants, the *rdpe* gene fragment of pMAR was replaced by PCR products amplified with primers MA135-MA144 (truncation mutants), MA145-MA172 (alanine scanning mutants and arginine substitution mutants). RDPE lacking hydrophobic domains was constructed using overlap extension PCR with primers MA125-MA134, and the products were cloned into pMA5.

For construction of the mCherry fluorescent protein fusions with the N-terminus of RDPE, the *mCherry* gene was amplified from the plasmid pDL-mCh, which was kindly provided by Dr. Ran Tu from TIB CAS, and cloned into the plasmid pMA5, resulting in plasmid pMAmCh. To separate the domains of mCherry and RDPE, a flexible linker (GGGGS)_2_ was introduced between the mCherry reporter protein and the nucleotide sequence of RDPE. Subsequently, the N-terminal fragments and full-length RDPE were amplified with MA175-MA184 and MA186/MA187, respectively, and cloned into the plasmid pMAmCh, resulting in nine corresponding recombinant plasmids. For construction of the RDPE derivative with a C-terminal 6-His tag (RDPE-H), the *rdpe* gene was amplified from the pMAR plasmid with the primer pair MA186 and MA188, which contained a 6-His tag coding sequence, and cloned into the pMA5 plasmid, resulting in plasmid pMAR-H. Similarly, for construction of the PhoD-H plasmid containing a C-terminal 6-His tag, the *phoD* gene was amplified from *B. subtilis* 168 chromosomal DNA using primers MA189 and MA190, and cloned into the pMA5 plasmid, resulting in plasmid pMAP-H. For 16 cytoplasmic proteins overproduced in *B. subtilis* 1A751, the corresponding genes were amplified from the *B. subtilis* 168 chromosomal DNA using the corresponding primers MA191-MA220. The amplified products were ligated into the linear vector backbone of pMAR amplified with primers MA123 and MA124, resulting in 16 corresponding recombinant plasmids. For construction of GFP fusions with several non-classically secreted proteins, a flexible linker (GSGGGS)_1_ was introduced between the nucleotide sequence of the non-classically secreted proteins and GFP. The *gfp* gene was amplified from the pDG plasmid using the primer pair MA221 and MA222 and cloned into the pMA5 plasmid, resulting in plasmid pMA5GFP. The genes *pdhD, rocF, ycgN, yisN* and *rdpe* were amplified using corresponding genomic DNA or plasmids as the templates with the corresponding primers MA223–MA232, which contain the nucleotide sequence of the flexible linker. The amplified products were ligated to the linear vector backbone of pMA5GFP, resulting in corresponding plasmids. For construction of GFP fusions with the N-terminus of RDPE, the N-terminal fragments N224 and N254 were amplified with MA231, MA233 and MA234, and cloned into pMA5GFP, resulting in corresponding plasmids.

A strain 1A700 deletion of two genes *lytC* and *lytD* was constructed using a marker-free multiple mutation system, which carries an AraR repressor and the *ara* promoter, as described previously^59^. To construct *B. subtilis* Δ*lytC*, three regions upstream (UP), downstream (DN) and deletion target region (G) of the *lytC* gene were generated using primers MA021 and MA 022, MA023 and MA024, and MA025 and MA026, respectively. The *cat* fragment was amplified from the plasmid pDG using primers MA027 and MA028. The *araR* gene was amplified from the *B. subtilis* 168 chromosomal DNA using the primers MA029 and MA030. The five fragments, which overlapped one another by 25 bp, were ligated by overlap extension PCR to obtain a 4.9 kb fragment. The fragment was transformed into *B. subtilis* 1A75S1, to yield the 1A75S2 strain through double crossover recombination between the disrupted *lytC* region of the DNA fragment and the chromosomal *lytC* gene. Finally, the *B. subtilis* Δ*lytC* was obtained by homologous recombination between two homologous DN fragments of the *lytc* gene. Similarly, the *lytD* gene was deleted and the *B. subtilis* Δ*lytC* Δ*lytD* (1A700) was obtained.

### Enzyme assays

The enzyme activity of RDPE in the cytoplasm and supernatant was determined as described previously^29^. Briefly, one millilitre of reaction mixture contained 20 g L^−1^ D-fructose in Tris/HCl buffer (50 mM, pH 8.0) and 200 μL culture medium or cell lysate supernatant of bacteria. The reactions were performed at 55 °C for 10 min and were stopped by boiling at 100 °C for 5 min. The generated D-psicose was analysed by a high-performance liquid chromatography (HPLC) system. One unit of enzyme activity is defined as the amount of enzyme that catalysed the formation of 1 μmol of D-psicose per minute at pH 8.0 and 55 °C.

### Vesicle isolation

Vesicles were isolated with minor modifications to the method described previously^60^. Briefly, bacterial cultures were centrifuged at 4,000 × g for 20 min at 4 °C, and the supernatant was collected and again centrifuged at 10,000 × g for 20 min at 4 °C to remove cellular debris. The resulting supernatant was concentrated using an Amicon ultrafiltration system (100 kDa). The concentrate was again centrifuged as described above to remove cellular debris. The resulting supernatant was then centrifuged at 100,000 × g for 2 h at 4 °C, and vesicle pellets were washed three times with phosphate buffered saline (PBS). Vesicles were resuspended in PBS for analysis.

### Cell fractionation

The culture medium was harvested by centrifugation at 4,000 × g at 4 °C for 10 min, and used as a cell supernatant fraction. The residual whole cell pellets, normalized to the same absorbance reading at OD_600_, were resuspended in lysis buffer (50 mM Tris/HCl, 200 mM NaCl, 2 mM EDTA, 1 mg ml^−1^ lysozyme and 100 mM dithiothreitol [DTT], pH 8.0). An aliquot of the cell suspension was incubated at 37 °C for 30 min. The pellets were further lysed using ultrasound sonication on ice for 5 min with a 5 s interval. Lysates were centrifuged at 4,000 × *g* for 10 min at 4 °C, yielding the pellet and soluble fractions. The fractions were mixed with 5× reducing SDS sample buffer with added 5% (W/V) β-mercaptoethanoland boiled for 10 min before being analysed using sodium dodecyl sulphate-polyacrylamide gel electrophoresis (SDS-PAGE).

### BN-PAGE

The oligomeric state of soluble recombinant RDPE was analysed by blue native polyacrylamide gel electrophoresis (BN-PAGE). The soluble protein fractions in the cytoplasm and culture supernatant were solubilized in 5× non-reducing sample buffer, and the samples were loaded onto acrylamide native gels stained with Coomassie blue. The running buffer and native PAGE gel were prepared as described previously^37^. NativeMarker^TM^ Unstained Protein Standard (Invitrogen Life Technologies, USA) was used as a molecular weight marker.

### Chemical cross-linking

*B. subtilis* 1A75R-H strain expressing protein RDPE-H with a C-terminal 6-His tag was cultured in 30 mL SR for 36 h. Cell aliquots (C1) and supernatant (S1) were collected for RDPE-H analysis. Cells were harvested by centrifuging at 4,000 × g for 10 min at 4 °C and washed three times with ice-cold PBS (pH8.3) to remove culture medium and proteins from the cells, then resuspended in PBS until an OD_600_ of 8.0 was reached. Then, add the DSS solution to a final concentration of 0.5 mM and the samples were incubated for 20 min at 37 °C. Aliquots of cells were collected to confirm that the crosslinker successfully cross-linked the intracellular RDPE-H (C2). After incubation, the cells were harvested and washed three times with 30 mL fresh SR medium. The cells were recovered for 1 h at 37 °C with rotation. The cell (C3) and supernatant (S3) fractions of recovered bacteria were analysed by 10% SDS-PAGE followed by anti-His tag immunoblotting. Similarly, the PhoD-H protein was cross-linked.

### Immunoblotting

Samples were prepared as described previously and analysed by SDS-PAGE for immunoblotting. Proteins were separated on 10% Bis-Tris Gel and transferred to nitrocellulose membranes. The blots were first probed with mouse primary antibodies and then with the anti-mouse IgG (H + L) secondary antibody. Detection was performed using CD/DAB substrate kit (Thermo Scientific, USA).

### Fluorescence measurements

The fluorescence intensity of fusion proteins was measured using a Multimode microplate reader (SpectraMax M5). The strains were grown in SR liquid medium at 37 °C for 48 h. The culture medium was harvested by centrifugation at 4,000 × g at 4 °C for 10 min. The cells were resuspended with equal volume of PBS (pH 8.0) buffer. For mCherry fused proteins, the extinction and emission wavelength were set at 532 and 610 nm, respectively. For GFP fused proteins, the extinction and emission wavelength were set at 488 and 520 nm, respectively. Mean fluorescence measurements were obtained from three separate experiments.

### Fluorescence microscope

All strains were grown in SR liquid medium at 37 °C to the mid-exponential phase. The cells were collected by centrifugation at 4,000 × g at 4 °C for 10 min, and were washed three times with water to remove culture medium from the cells. Fluorescent microscopy was performed with Leica DM5000 B Upright microscopy.

## Additional Information

**How to cite this article:** Zhao, L. *et al*. Multimer recognition and secretion by the non-classical secretion pathway in *Bacillus subtilis. Sci. Rep.* **7**, 44023; doi: 10.1038/srep44023 (2017).

**Publisher's note:** Springer Nature remains neutral with regard to jurisdictional claims in published maps and institutional affiliations.

## Supplementary Material

Supplementary Information
