# Supplementary material for: Multimer recognition and secretion by the non-classical secretion pathway in Bacillus subtilis
Source: Sci Rep. 2017 Mar 9;7:44023. doi: 10.1038/srep44023 (PMC5343618; doi:10.1038/srep44023)
Supplement: Supplementary Information [file srep44023-s1.pdf]

Multimer recognition and secretion by the non-classical secretion pathway in *Bacillus subtilis*

Liuqun Zhao[1,2†], Jingqi Chen[1,2†], Jibin Sun[1,2], Dawei Zhang[1,2,3*]

[1]Tianjin Institute of Industrial Biotechnology, Chinese Academy of Sciences, Tianjin 300308, P. R. China

[2]Key Laboratory of Systems Microbial Biotechnology, Chinese Academy of Sciences, Tianjin 300308, P. R. China

[3]National Engineering Laboratory for Industrial Enzymes, Tianjin 300308, P. R. China

[†] These authors contributed equally to this work.

*Corresponding authors. E-mail: zhang_dw@tib.cas.cn; Tel: +86-22-24828749; Fax: +86-22-24828749

SUPPLEMENTARY DATA

Supplementary Table S1: Strains and plasmids used in this study.

Strain or plasmid	Description	Source
Strains		
E. coli		
DH5α	F^- Φ80 Δ*lac* ΔM15 Δ(*lacZYA argF*)*U169 deoR recA1 endA1 hsdR17* (rK mK) *supE44* λ^- *thi-1 gyrA96 relA1*	Lab stock
B.subtilis		
168	*trpC2*	Lab stock
1A751	*trpC2 egl*SΔ102 *bgl*T/*bgl*SΔEV *apr*E *npr*E *his*	BGSC, US
1A75S1	1A751::Δ*araR*::P*ara*-Spe; Spe^r	This work
1A75S2	1A751:: Δ*araR*::P*ara*-Sp:: *lytC* (DN)-*cat-araR*; Cm^r	This work
1A75S3	1A751:: Δ*araR*::P*ara*-Sp:: *lytD* (DN)-*cat-araR*; Cm^r	This work
1A700	1A751:: Δ*araR*::P*ara*-Sp Δ*lytClytD*; Spe^r	This work
Plasmids		
pHCMC05	Overexpression vector, P*spac*, Ap^r, Cm^r	Lab stock
pDL-mCh	pDL derivative, Cm^r; m*Cherry*	Lab stock
pDG	pDL derivative, Cm^r; *gfp*	Lab stock
pMA5	*E. coli/B. subtilis* shuttle vector, $P_{Hpa\,II}$, Ap^r, Km^r	BGSC, US
pMAR	pMA5 derivative, *rdpe*	[1]
pMASR	pMA5 derivative, P_{spac} , Ap^r, Cm^r; *rdpe*	This work
pMARΔC1	pMAR derivative, delets 1 residue at the C terminus of RDPE	This work
pMARΔC2	pMAR derivative, delets 2 residue at the C terminus of RDPE	This work
pMARΔC3	pMAR derivative, delets 3 residue at the C terminus of RDPE	This work
pMARΔC5	pMAR derivative, delets 5 residue at the C terminus of RDPE	This work
pMARΔC7	pMAR derivative, delets 7 residue at the C terminus of RDPE	This work
pMARΔN1	pMAR derivative, delets 1 residue at the Nterminus of RDPE	This work
pMARΔN2	pMAR derivative, delets 2 residue at the Nterminus of RDPE	This work
pMARΔN3	pMAR derivative, delets 3 residue at the Nterminus of RDPE	This work
pMARΔN5	pMAR derivative, delets 5 residue at the Nterminus of RDPE	This work
pMARΔN7	pMAR derivative, delets 7 residue at the Nterminus of RDPE	This work
pMARΔHD1	pMAR derivative, delets HD1 of RDPE	This work
pMARΔHD2	pMAR derivative, delets HD2 of RDPE	This work
pMARΔHD3	pMAR derivative, delets HD3 of RDPE	This work
pMARΔHD4	pMAR derivative, delets HD4 of RDPE	This work
pMARL100A	pMAR derivative, L100A substitution	This work
pMARD102A	pMAR derivative, D102A substitution	This work
pMARI103A	pMAR derivative, I103A substitution	This work
pMARV106A	pMAR derivative, V106A substitution	This work
pMARG107A	pMAR derivative, G107A substitution	This work

pMARV175A	pMAR derivative, V175A substitution	This work
pMARV180A	pMAR derivative, V180A substitution	This work
pMARG181A	pMAR derivative, G181A substitution	This work
pMARV182A	pMAR derivative, V182A substitution	This work
pMARL184A	pMAR derivative, L184A substitution	This work
pMARV106R	pMAR derivative, V106R substitution	This work
pMARV175R	pMAR derivative, V175R substitution	This work
pMARL184R	pMAR derivative, L184R substitution	This work
pMARRv54	pMAR derivative, V175A/L184A double substitution	This work
pMARRv02	pMAR derivative, V180A/G181A/V182A triple substitution	This work
pMAR-H	pMAR derivative, 6 × His tag	This work
pMAP-H	pMA5 derivative, *phoD*, 6 × His tag	This work
pMA5mCh	pMA5 derivative, *mCherry*	This work
pMA5mCh1	pMA5mCh derivative, *rdpe-mCherry*	This work
pMA5mCh2	pMA5mCh derivative, *TMS*2- *mCherry*	This work
pMA5mCh3	pMA5mCh derivative, *TMS4-mCherry*	This work
pMA5mCh4	pMA5mCh derivative, *N101-mCherry*	This work
pMA5mCh5	pMA5mCh derivative, *N119-mCherry*	This work
pMA5mCh6	pMA5mCh derivative, *N160-mCherry*	This work
pMA5mCh7	pMA5mCh derivative, *N194-mCherry*	This work
pMA5mCh8	pMA5mCh derivative, *N224-mCherry*	This work
pMA5mCh9	pMA5mCh derivative, *N254-mCherry*	This work
pMA5CitH	pMA5 derivative, *citH*	This work
pMA5FusA	pMA5 derivative, *fusA*	This work
pMA5Eno	pMA5 derivative, *eno*	This work
pMA5FbaA	pMA5 derivative, *fbaA*	This work
pMA5GapA	pMA5 derivative, *gapA*	This work
pMA5GroEL	pMA5 derivative, *groel*	This work
pMA5KatA	pMA5 derivative, *katA*	This work
pMA5PdhA	pMA5 derivative, *pdhA*	This work
pMA5PdhB	pMA5 derivative, *pdhB*	This work
pMA5PdhD	pMA5 derivative, *pdhD*	This work
pMA5RocF	pMA5 derivative, *rocF*	This work
pMA5SodA	pMA5 derivative, *sodA*	This work
pMA5YceD	pMA5 derivative, *yceD*	This work
pMA5YcgN	pMA5 derivative, *ycgN*	This work
pMA5YwjH	pMA5 derivative, *ywjH*	This work
pMA5GFP	pMA5 derivative, *gfp*	This work
pMA5PdhD-GFP	pMA5 derivative, *pdhD-gfp*	This work
pMA5RocF-GFP	pMA5 derivative, *rocF-gfp*	This work
pMA5YcgN-GFP	pMA5 derivative, *ycgN-gfp*	This work
pMA5YisN-GFP	pMA5 derivative, *yisN-gfp*	This work
pMAR-GFP	pMA5 derivative, *rdpe-gfp*	This work

Supplementary Table S2: Oligonucleotides used in this study.

Name	Sequence 5' to 3'	Description	Source
**Primers for construction of *B. subtilis* strain 1A700**			
MA021	ATTCCGAATGAAATGCCTGCT	Δ*lytC*(UP)	This work
MA022	GTATCTATTGCTTCCCCCAAC	Δ*lytC*(UP)	This work
MA023	AAGAGTTGGGGGAAGCAATAGATACGGAGTAA AAACAGCTGCTTTC	Δ*lytC*(DN)	This work
MA024	AACTAATGGGTGCTTTAGTTGAAGAGAAGCTCG TCAATTCTCGTTC	Δ*lytC*(DN)	This work
MA025	GTCCGCACGAAAACTGAATGAATAAGGCACTG CTGTACAAATATCA	Δ*lytC*(G)	This work
MA026	CCGGTCTCTCGTTCCAAGAT	Δ*lytC*(G)	This work
MA027	CACTAACCTGCCCCGTTAGTTGAAGGCATTTTC TGTCAATGTTTTCTTAC	*cat*	This work
MA028	GAACTGGAATGAGATTGAGCCATTTATTCATTC AGTTTTCGTGCGGACTGGCG	*cat*	This work
MA029	TCTTCAACTAAAGCACCCATTAGTTC	*araR*	This work
MA030	CTTCAACTAACGGGGCAGGTTAGTG	*araR*	This work
MA031	CAAGACGGAATGCTGGTACAT	Δ*lytD*(UP)	This work
MA032	TTCCAACAGGACTTGACGTTC	Δ*lytD*(UP)	This work
MA033	ACAGGAACGTCAAGTCCTGTTGGAAATTACTAC GGAGCCAAATATGC	Δ*lytD*(DN)	This work
MA034	AACTAATGGGTGCTTTAGTTGAAGACCTATGCT TCGATCCCCTTT	Δ*lytD*(DN)	This work
MA035	GTCCGCACGAAAACTGAATGAATAAGCAAACA GCCATATGTCACC	Δ*lytD*(G)	This work
MA036	TCGGATTGCTGTCGTATGCT	Δ*lytD*(G)	This work
**Primers for construction of inducible expression vector pMASR**			
MA121	CCTCGCAGAGCACACACTTTATGAAGCTCAGGC	P_{spac}-*lacI* cassette	This work
MA122	TAATAAATACCATATTTCATCTAGAGGATCCTCA CCTCCTTAAGC	P_{spac}-*lacI* cassette	This work
MA123	ATGAAATATGGTATTTATTACGCTTATTGG	pMAR vector bone	This work
MA124	TTCATAAAGTGTGTGCTCTGC	pMAR vector bone	This work
**Primers for construction of deletion mutants**			
MA125	CCACCTAAAAAGGAGCGATTTACATATGAAATA TGGTATTTATTACGCTTATTGG	HD1(UP)	This work
MA126	ATCTATATAATATTTGTAATCTCCAT	HD1(UP)	This work
MA127	GATTACAAATATTATATAGATTCTTGCGGCGCTTT TTCTGAC	HD1(DN)	This work
MA128	GAGACGCTCCGGAAATTGAAGTCCAAACCTTTT	HD1(DN)	This work

MA129	CTTCAATTTCCGGAGCGTCT	HD2(UP)	This work
MA130	GCTTGAGCTCGACTCTAGAGGATCCTTAGACTT CAAATACATGTTTTAC	HD2(DN)	This work
MA131	TATGTCATATTCTTCAGCATACTG	HD3(UP)	This work
MA132	ATGCTGAAGAATATGACATAATGTTGAATACATG CGATGAAGCG	HD3(DN)	This work
MA133	TGAAGCGTTGGCATACGTTGAAGAGGAGGAAG	HD4(UP)	This work
MA134	CTCTTCAACGTATGCCAACG	HD4(DN)	This work
**Primers for construction of truncation mutants**			
MA135	GCTTGAGCTCGACTCTAGAGGATCCTTATTCAA ATACATGTTTTACAAAG	RDPEΔC1	This work
MA136	GCTTGAGCTCGACTCTAGAGGATCCTTAAAATA CATGTTTTACAAAGTGC	RDPEΔC2	This work
MA137	TAAGGATCCTCTAGAGTCGAG	RDPEΔC3	This work
MA138	CTCGACTCTAGAGGATCCTTATTTTACAAAGTG CAACGCAC	RDPEΔC5	This work
MA139	GCTTGAGCTCGACTCTAGAGGATCCTTAAAAGT GCAACGCACTTTGAG	RDPEΔC7	This work
MA140	ACCTAAAAAGGAGCGATTTACATATGTATGGTAT TTATTACGCTTAT	RDPEΔN1	This work
MA141	ACCTAAAAAGGAGCGATTTACATATGATTTATTA CGCTTATTGGGAA	RDPEΔN2	This work
MA142	TAAAAAGGAGCGATTTACATATGTACGCTTATTG GGAAAAGGAAT	RDPEΔN3	This work
MA143	CATATGTAAATCGCTCCTTTTTAG	RDPEΔN5	This work
MA144	ACCTAAAAAGGAGCGATTTACATATGTATTGGG AAAAGG	RDPEΔN7	This work
**Primers for construction of alanine scanning mutants and arginine substitution mutants**			
MA145	GGAAATTGAAGGCAATGGATATTCAT	RDPEL100A	This work
MA146	ATGAATATCCATTGCCTTCAATTTCC	RDPEL100A	This work
MA147	GAAGTTAATGGCTATTCATATTGTTGG	RDPED102	This work
MA148	CCAACAATATGAATAGCCATTAACTTC	RDPED102	This work
MA149	GAAGTTAATGGATGCTCATATTGTTGG	RDPEI103	This work
MA150	CCAACAATATGAGCATCCATTAACTTC	RDPEI103	This work
MA151	ATTCATATTGCTGGAGGCGCACTCTAT	RDPEV106A	This work
MA152	ATAGAGTGCGCCTCCAGCAATATGAAT	RDPEV106A	This work
MA153	ATTCATATTGTTGCAGGCGCACTCTAT	RDPEG107A	This work
MA154	ATAGAGTGCGCCTGCAACAATATGAAT	RDPEG107A	This work
MA155	CATACGTTGAAGAGGCTGGCTCTTCTAATGT	RDPEV175A	This work
MA156	ACATTAGAAGAGCCAGCCTCTTCAACGTATG	RDPEV175A	This work
MA157	TTGGCTCTTCTAATGCTGGTGTTATGTTAG	RDPEV180A	This work
MA158	CTAACATAACACCAGCATTAGAAGAGCCAA	RDPEV180A	This work
MA159	GCTCTTCTAATGTTGCTGTTATGTTAGAT	RDPEG181A	This work
MA160	ATCTAACATAACAGCAACATTAGAAGAGC	RDPEG181A	This work

MA161	CTTCTAATGTTGGTGCTATGTTAGATACTT	RDPEV182A	This work
MA162	AAGTATCTAACATAGCACCAACATTAGAAG	RDPEV182A	This work
MA163	AATGTTGGTGTTATGGCAGATACTTTTCACAT	RDPEL184A	This work
MA164	ATGTGAAAAGTATCTGCCATAACACCAACATT	RDPEL184A	This work
MA165	ATTCATATTCGTGGAGGCGCACTCTAT	RDPEV106R	This work
MA166	ATAGAGTGCGCCTCCACGAATATGAAT	RDPEV106R	This work
MA167	CATACGTTGAAGAGCGTGGCTCTTCTAATGT	RDPEV175R	This work
MA168	ACATTAGAAGAGCCACGCTCTTCAACGTATG	RDPEV175R	This work
MA169	AATGTTGGTGTTATGCGAGATACTTTTCACAT	RDPEL184R	This work
MA170	ATGTGAAAAGTATCTCGCATAACACCAACATT	RDPEL184R	This work
MA171	CTTCTAATGCTGCTGCTATGTTAGATACTT	Rv54	This work
MA172	AAGTATCTAACATAGCAGCAGCATTAGAAG	Rv54	This work
**Primers for construction of mCherry and its fusion proteins expression vectors**			
MA173	CCACCTAAAAAGGAGCGATTTACATATGGTGAG CAAGGGCGAGG	*mCherry*	This work
MA174	TTGAGCTCGACTCTAGAGGATCCCTACTTGTAC AGCTCGTCCAT	*mCherry*	This work
MA175	CCACCTAAAAAGGAGCGATTTACATATGTTAAT GGATATTCATATTGTTG	HD2-mCh	This work
MA176	CCTCCTCGCCCTTGCTCACCATATAATCTACAGG CCAATAACC	HD2-mCh	This work
MA177	CCACCTAAAAAGGAGCGATTTACATATGGTTGG CTCTTCTAATGTTG	HD4-mCh	This work
MA178	CTCCTCGCCCTTGCTCACCATTATATTCATGTGA AAAGTATCTAAC	HD4-mCh	This work
MA179	GCCACCTCCGCCTGAACCGCCTCCACCTGACAT TAACTTCAATTTCCGGAGC	N101-mCh	This work
MA180	GCCACCTCCGCCTGAACCGCCTCCACCTGAGG AATAATCTACAGGCCAATAAC	N119-mCh	This work
MA181	GCCACCTCCGCCTGAACCGCCTCCACCTGAATA GCCTTCAAAACGGTTAAGAAC	N160-mCh	This work
MA182	GCCACCTCCGCCTGAACCGCCTCCACCTGAATC TTCCTCTATATTCATGTGAAAAG	N194-mCh	This work
MA183	GCCACCTCCGCCTGAACCGCCTCCACCTGACAT ACCTTTTCCTGGTACTTTACG	N224-mCh	This work
MA184	GCCACCTCCGCCTGAACCGCCTCCACCTGATGT	N254-mCh	This work
MA185	GGCGGTTCAGGCGGAGGTGGCTCTGGCGGTGG	Linker	This work
MA186	GCCACCTCCGCCTGAACCGCCTCCACCTGAGA CTTCAAATACATGTTTTACAAAGTGC	*rdpe*	This work
MA187	CCACCTAAAAAGGAGCGATTTACATATGAAATA	*rdpe*	This work
**Primers for construction of RDPE-H /PhoD-H with a C-terminal 6-Histag expression vector**			
MA188	TTAGTGATGATGATGATGATGGACTTCAAATACA TGTTTTACAAAG	RDPE-H	This work
MA189	CCACCTAAAAAGGAGCGATTTACATATGGCATA	PhoD-H	This work

MA190	TCCTTAGTGATGATGATGATGATGATTCGTGATT TTTGCACGCTT	PhoD-H	This work
**Primers for construction of 16 cytoplasmic proteins expression vectors**			
MA191	CCACCTAAAAAGGAGCGATTTACATATGCTTGC CATACTCGGTTTTG	*citH*	This work
MA192	TTGAGCTCGACTCTAGAGGATCCTTAGAAAGAA	*citH*	This work
MA193	CCACCTAAAAAGGAGCGATTTACATATGGCAAG AGAGTTCTCCTT	*fusA*	This work
MA194	TTGAGCTCGACTCTAGAGGATCCTTATTCGCCTT	*fusA*	This work
MA195	CCACCTAAAAAGGAGCGATTTACATATGCCATA CATTGTTGATGTTTATG	*eno*	This work
MA196	TTGAGCTCGACTCTAGAGGATCCTTACTTGTTTA AGTTGTAGAAAG	*eno*	This work
MA197	CCACCTAAAAAGGAGCGATTTACATATGCCTTT AGTTTCTATGACG	*fbaA*	This work
MA198	TTGAGCTCGACTCTAGAGGATCCTTAAGCTTGG TTTGAAGAAC	*fbaA*	This work
MA199	CCACCTAAAAAGGAGCGATTTACATATGGCAGT AAAAGTCGGTATTAAC	*gapA*	This work
MA200	TTGAGCTCGACTCTAGAGGATCCTTAAAGACCT TTTTTTGCGATG	*gapA*	This work
MA201	CCACCTAAAAAGGAGCGATTTACATATGGCAAA AGAAATTAAGTTTAG	*groel*	This work
MA202	TTGAGCTCGACTCTAGAGGATCCTTACATCATTC CACCCATAC	*groel*	This work
MA203	CTAAAAAGGAGCGATTTACATATGAGTTCAAAT AAACTGACAAC	*katA*	This work
MA204	TTGAGCTCGACTCTAGAGGATCCTTAAGAATCT TTTTTAATCGG	*katA*	This work
MA205	CTAAAAAGGAGCGATTTACATATGGCTGCAAAA ACGAAAAAAGCTATCG	*pdhA*	This work
MA206	TTGAGCTCGACTCTAGAGGATCCTTACTTCGAC TCCTTCTGT	*pdhA*	This work
MA207	CTAAAAAGGAGCGATTTACATATGGCGCAAATG ACAATGAT	*pdhB*	This work
MA208	TTGAGCTCGACTCTAGAGGATCCTTAAAATTCA AGCACTTTTC	*pdhB*	This work
MA209	CTAAAAAGGAGCGATTTACATATGGTAGTAGGA GATTTCC	*pdhD*	This work
MA210	TTGAGCTCGACTCTAGAGGATCCTTATTTTACGA	*pdhD*	This work
MA211	CTAAAAAGGAGCGATTTACATATGGATAAAACG ATTTCGGT	*rocF*	This work
MA212	TTGAGCTCGACTCTAGAGGATCCTTACAGCAGC TTCTTCC	*rocF*	This work

MA213	CTAAAAAGGAGCGATTTACATATGGCTTACGAACTTCCAG	*sodA*	This work
MA214	TTGAGCTCGACTCTAGAGGATCCTTATTTTGCTTCGCTGTAT	*sodA*	This work
MA215	CTAAAAAGGAGCGATTTACATATGACAATTTCATTGGCAAAAGG	*yceD*	This work
MA216	TTGAGCTCGACTCTAGAGGATCCTTAACCGACTTGCAAACC	*yceD*	This work
MA217	CTAAAAAGGAGCGATTTACATATGACAACACCTTACAAACACGAGC	*ycgN*	This work
MA218	TTGAGCTCGACTCTAGAGGATCCTTAGAACATTTCACTGATTG	*ycgN*	This work
MA219	CTAAAAAGGAGCGATTTACATATGTTATTCTTTGTTGATAC	*ywjH*	This work
MA220	TTGAGCTCGACTCTAGAGGATCCTTATTTGTTCCAGTCTGC	*ywjH*	This work
**Primers for construction of GFP fusion proteins expression vectors**			
MA221	GGTAGCGGTGGAGGTGGCAGCATGAGTAAAGGAGAAGAACTTTT	*gfp*	This work
MA222	AGCTCGACTCTAGAGGATCCCTATTTGTATAGTTCATCCATGC	*gfp*	This work
MA223	CTAAAAAGGAGCGATTTACATATGGTAGTAGGAGATTTCC	*pdhD*	This work
MA224	GCTGCCACCTCCACCGCTACCTTATTTTACGATGTGAATCG	*pdhD*	This work
MA225	CTAAAAAGGAGCGATTTACATATGGTAGTAGGAGATTTCC	*rocF*	This work
MA226	GCTGCCACCTCCACCGCTACCCAGCAGCTTCTTCCCTAACAG	*rocF*	This work
MA227	CTAAAAAGGAGCGATTTACATATGACAACACCTTACAAACACG	*ycgN*	This work
MA228	GCTGCCACCTCCACCGCTACCGAACATTTCACT	*ycgN*	This work
MA229	CTAAAAAGGAGCGATTTACATATGAAGAAAAAGGAGACAGCAT	*yisN*	This work
MA230	CTGCCACCTCCACCGCTACCTTTGCGCCCCCGTTGGACT	*yisN*	This work
MA231	CCACCTAAAAAGGAGCGATTTACATATGAAATATGGTATTTATTACGCTTATTGG	*rdpe*	This work
MA232	GCTGCCACCTCCACCGCTACCGACTTCAAATACATGTTTTACAAAG	*rdpe*	This work
MA233	ATGCTGCCACCTCCACCGCTACCCATACCTTTTCCTGGTACTTTAC	N224-GFP	This work
MA234	ATGCTGCCACCTCCACCGCTACCTGTTCCTCCCTGCATTACAAAT	N254-GFP	This work

Supplementary figures

Supplementary Figure S1: Secretion of RDPE in strain WB600 which is deficient in six extracellular proteases. SDS-PAGE analysis of RDPE protein in the whole cell lysates (WCL) and culture supernatant (SN) fractions with 48 h incubation from the strains 1A751(a) and WB600(b). The band of RDPE (33 kDa) is labeled with an arrow.

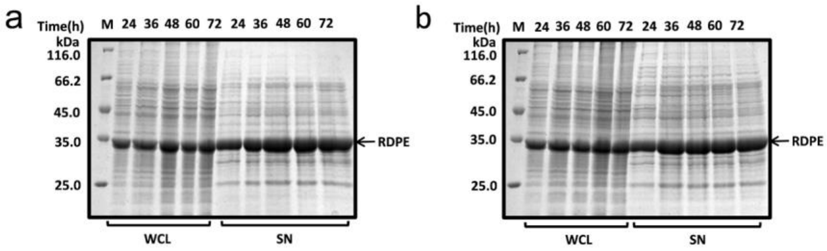

Supplementary Figure S2: Bioinformatics analysis of RDPE. (a) The hydropathy analysis of RDPE using the scale **Hphob. / Kyte & Doolittle**. The six extremely hydrophobic regions are indicated by Arabic numerals. (b) Transmembrane regions are predicted by the SPLIT server. The red line indicates transmembrane helix preference. ■ Four potential transmembrane segments HD1, HD2, HD3 and HD4 positions are indicated. (c) Alignment of the amino acid sequences of DPEase family enzymes. Sequences for DPEase from *Ruminococcus sp.* 5_1_39BFAA (RDPE; GenBank accession no: ZP04858451) were aligned with those DPEase from *Clostridium bolteae* ATCC BAA-613 (CBRDPE; EDP19602.1), *Agrobacterium tumefaciens str*. C58 (ATRDPE; AAK88700.1), *Agrobacterium fabrum* str. C58 (AFRDPE; AAK88700.1), *Clostridium cellulolyticum* H10 (CCRDPE; ACL75304) and *Clostridium scindens* ATCC 35704 (CSRDPE; ZP_02432283.1). The sequence alignment performed using the Clustal Omega and the secondary structure predicted by the Phyre2[2] were displayed using ESPript 3.0[3]. ■ The residues are highly conserved. ■ The residues are identical. Sequence numbering is based on RDPE, and its secondary structure elements are shown for its corresponding sequences.

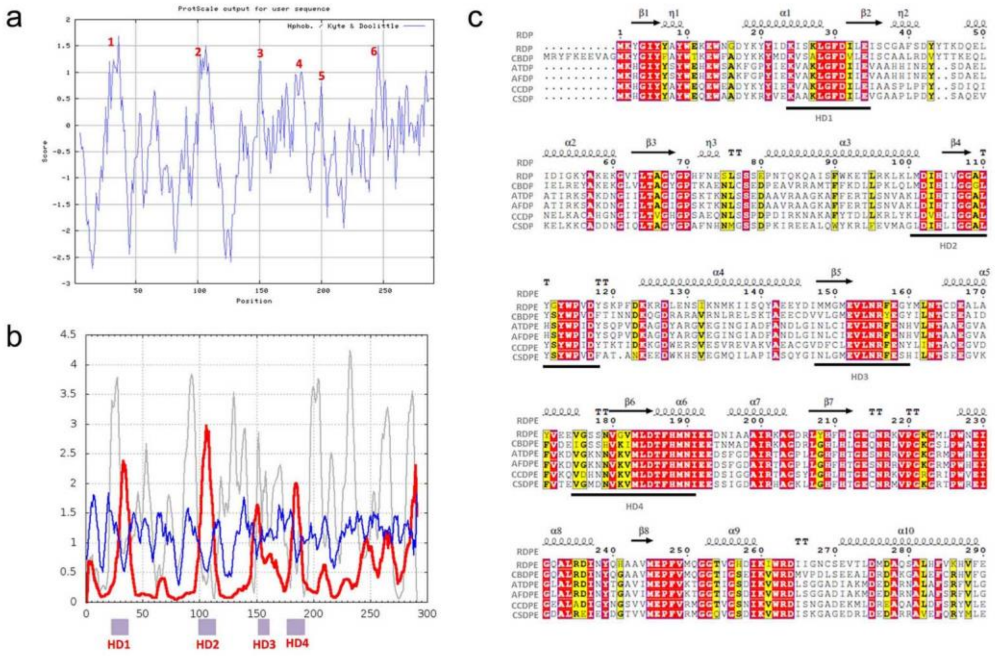

Supplementary Figure S3: Alanine scanning mutagenesis in two hydrophobic domains of RDPE. All residues of two transmembrane segments HD2 (from L100 to Y118) and HD4 (from V175 to I191) were changed to alanine except for the residue Ala^{109}, which is an intrinsic alanine at this site. The indicated strains expressing alanine scanning mutated protein were grown in SR medium at 37 ℃ for 48 h. SDS-PAGE analysis of the whole cell lysates (WCL) and culture supernatant (SN) fractions of single amino acid mutations of RDPE. The band of RDPE (33 kDa) is labeled with an arrow.

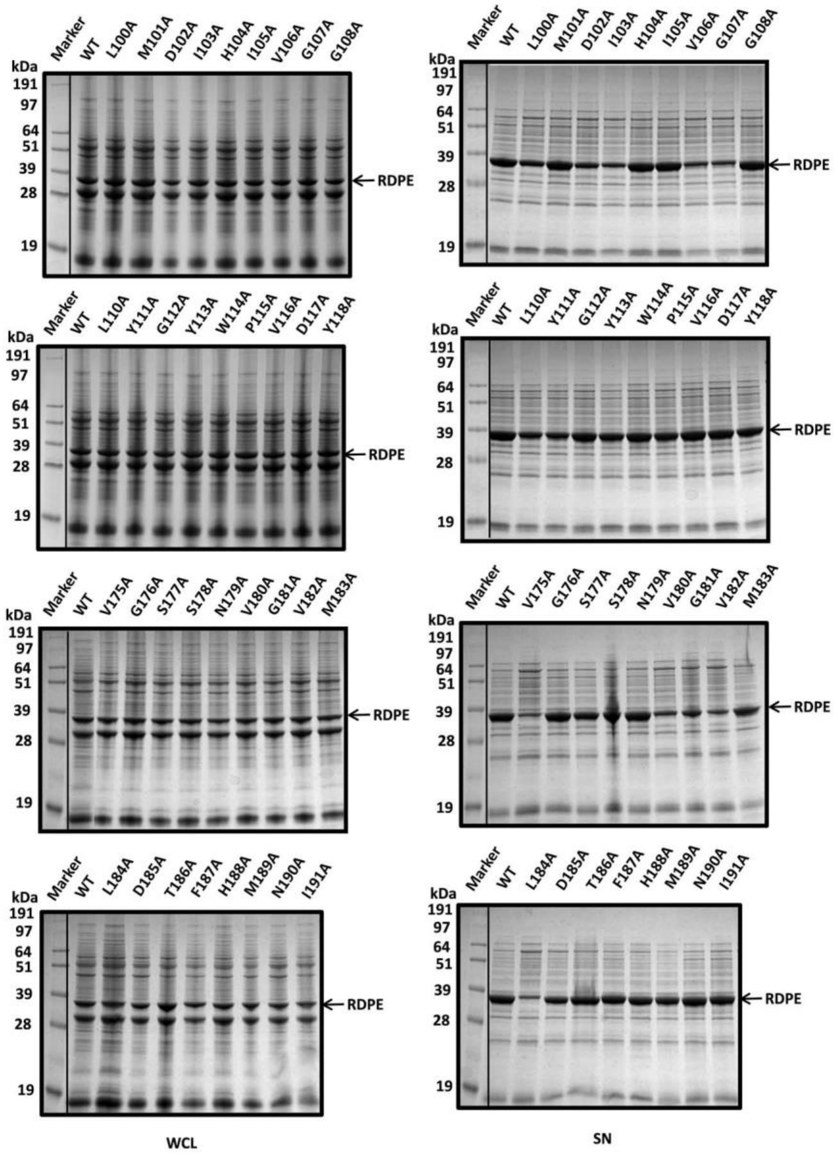

Supplementary Figure S4: Stability of RDPE secretion-disrupting variants in the culture supernatant. The soluble cell extracts were added to the culture after 48 h of growth. The samples at different times 0 min (a) and 120 min (b) after addition of whole-cell extracts were analysed by SDS-PAGE. The control line contains control sample without addition of crude extracts. The remaining lanes contain samples at 120 min after addition of whole-cell extracts.

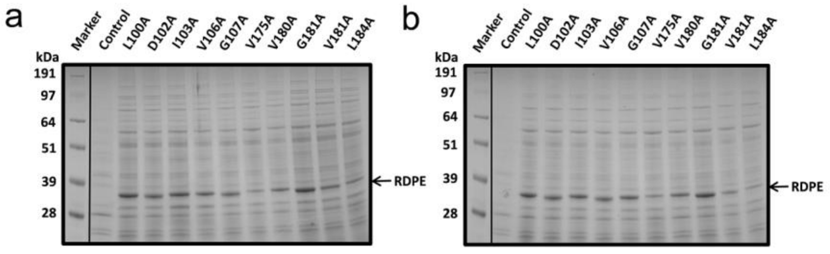

Supplementary Figure S5: Translocation of fusion mCherry/GFP proteins by non-classical secretion pathway. (a) Domain structure of the fusion mCherry proteins. (b) Fluorescence measurement of fusion mCherry proteins in the cells. Cells were collected after 48 h cultivation and resuspended with equal volume of PBS (pH 8.0) buffer. The extinction and emission wavelength were set at 532 and 610 nm, respectively. Error bars are SDs from three independent experiments. (c) The fusion proteins N101-mCh, N119-mCh, N160-mCh and N194-mCh in the cytoplasmic fraction (CP) and in the culture supernatant (SN) with 24 incubation were analysed by BN-PAGE. No band was detected under the nondenaturing condition. (d) Fluorescence measurement of fusion GFP proteins in the cells. Cells were collected after 48 h cultivation and resuspended with equal volume of PBS (pH 8.0) buffer. The extinction and emission wavelength were set at 488 and 520 nm, respectively. Error bars are SDs from three independent experiments.

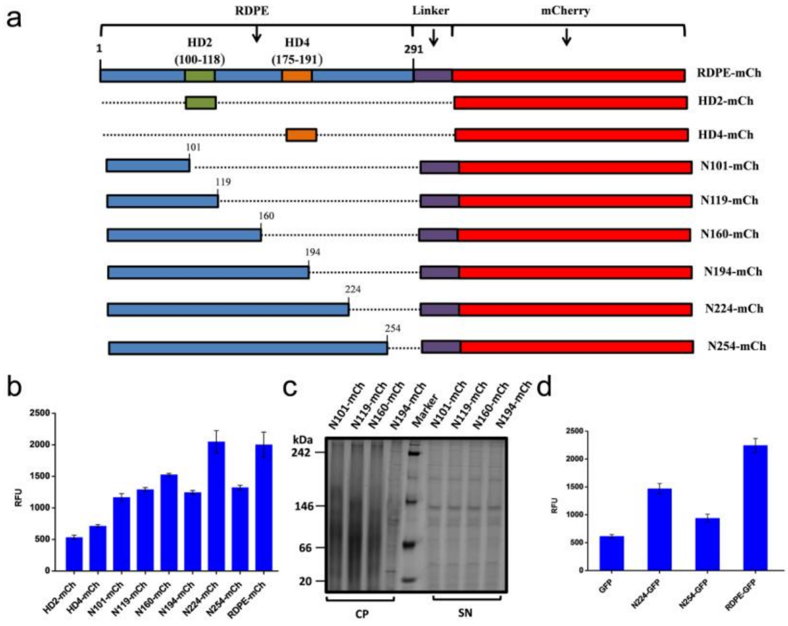

Supplementary Figure S6: The expression and secretion of cytoplasmic proteins in *B. subtilis*. SDS-PAGE analysis of the whole cell lysates (WCL) and culture supernatant (SN) fractions of *B. subtilis* 1A751 containing the plasmids expressing various cytopalsmic proteins after 48 h cultivation. The corresponding bands of FusA (76.0 kDa), Eno (46.0 kDa), FbaA (30.0 kDa), GapA (35.0 kDa), GroEL (57.0 kDa), KatA (54.0 kDa), PdhA (41.0 kDa), PdhD (49.0 kDa), RocF (32.0 kDa), SodA (22.0 kDa), YceD (20.0 kDa), YcgN (56.0 kDa) and YwjH (22.0 kDa) are labeled with arrows.

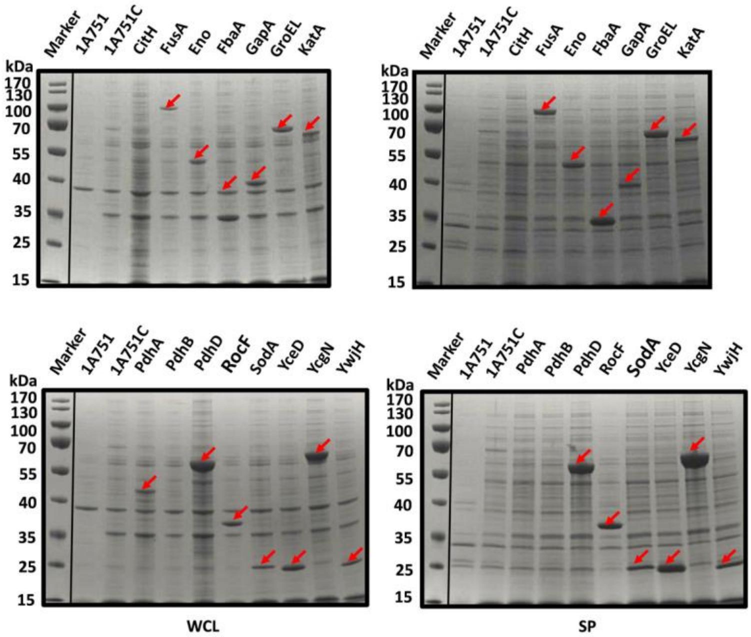

	FusA	BN-PAGE kDa expected (+/- 15%)	Eno	BN-PAGE kDa expected (+/- 15%)	FbaA	BN-PAGE kDa expected (+/- 15%)	GapA	BN-PAGE kDa expected (+/- 15%)	GroEL	BN-PAGE kDa expected (+/- 15%)	KatA	BN-PAGE kDa expected (+/- 15%)	PdhD	BN-PAGE kDa expected (+/- 15%)	RocF	BN-PAGE kDa expected (+/- 15%)	SodA	BN-PAGE kDa expected (+/- 15%)	YceD	BN-PAGE kDa expected (+/- 15%)	YcgN	BN-PAGE kDa expected (+/- 15%)	YwjH	BN-PAGE kDa expected (+/- 15%)
Monomer	76	65-87	46	39-53	30	25-35	35	30-40	57	48-66	54	46-62	49	42-56	32	27-37	22	19-25	20	17-23	56	48-64	22	19-25
Dimer									114	97-131			98	83-113			44	37-51	40	34-46				
Trimer							105	89-121																
Tetramer											216	184-248									224	190-258		
Pentamer					150	128-172									160	136-184								
Heptamer																							154	131-177

Supplementary Figure S7: Predicted masses of homologous non-classically secreted proteins oligomeric forms and corresponding masses expected by BN-PAGE analysis.

Supplementary Figure S8: The C- and N- terminal sequences are insufficient to direct the recombinant GFP translocate across cell membrane. The whole cell lysates (WCL) and culture supernatant (SN) fractions with 48 h incubation were analysed by SDS-PAGE. GFP is the strain expressing wild-type GFP protein. C20-GFP is the strain expressing recombinant GFP fused with C20 sequence. N20-GFP is the strain expressing recombinant GFP fused with N20 sequence. The parent strain 1A751 is regarded as a negative control. Corresponding bands are labeled with arrows.

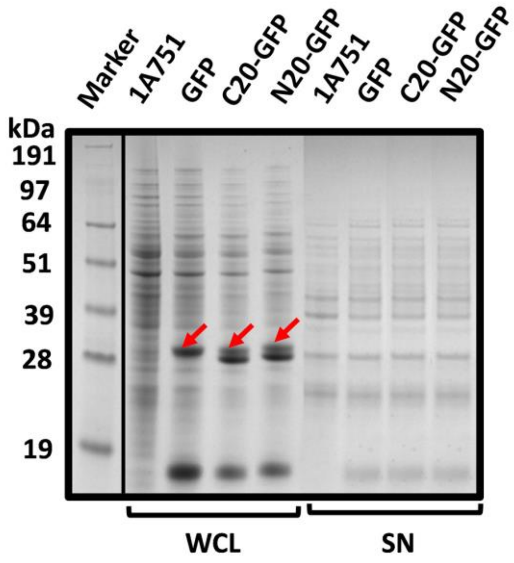

Supplementary Figure S9: Predicted tertiary structure of homotetramer RDPE. The structure was generated based on the template of D-Psicose 3-epimerase from *Clostridium cellulolyticum* H10 (PDB ID: 3VNK) using the HOMCOS server[4]. The subunits A, B, C, and D are shown in different colors. The segments HD2 (a) and HD4 (b) are indicated. The residues TRY118 and ILE191 in subunit A are shown using the stick molecular representation. The left panel shows the region near the residues TRY118 and ILE191. The distance between two residues TRY118/ILE191 in subunits A and B is presented for the model.

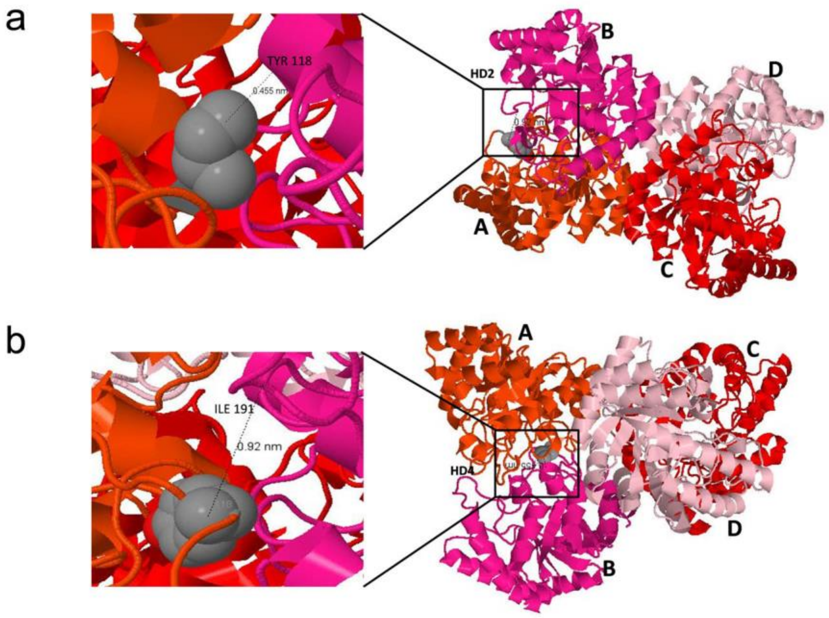

Supplementary Figure S10: Secretion of RDPE in the strain with deficiency in EXS system. 1A751R (expressing RDPE), Δ*yukDC*R (lacking *yukDC* and expressing RDPE), Δ*yukBA*R (lacking *yukBA* and expressing RDPE), Δ*yueB*R (lacking *yueB* and expressing RDPE) and Δ*yueCD*R (lacking *yueCD* and expressing RDPE), were grown in SR medium at 37 ℃ for 48 h. The whole cell lysates (WCL) and culture supernatant (SN) fractions fractions were prepared and analysed by SDS-PAGE. The parent strain 1A751 and 1A751C harboring empty vector pMA5 are regarded as negative controls. The band of RDPE (33 kDa) is labeled with an arrow.

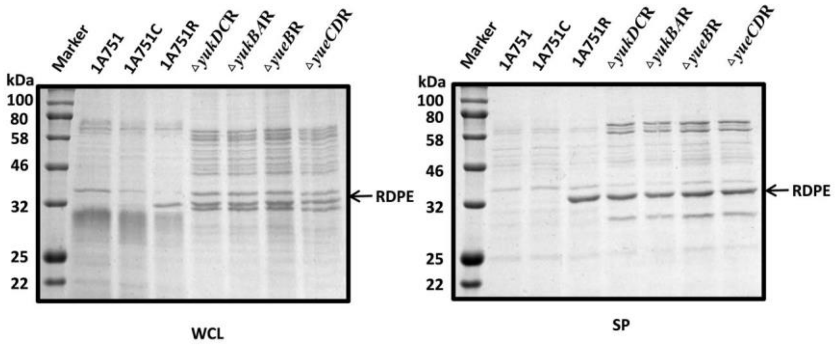

References

1 Chen, J. *et al.* A novel strategy for protein production using non-classical secretion pathway in Bacillus subtilis. *Microbial cell factories* **15**, 69, doi:10.1186/s12934-016-0469-8 (2016).

2 Kelley, L. A., Mezulis, S., Yates, C. M., Wass, M. N. & Sternberg, M. J. E. The Phyre2 web portal for protein modeling, prediction and analysis.

3 Robert, X. & Gouet, P. Deciphering key features in protein structures with the new ENDscript server. *Nucleic acids research* **42**, W320-324, doi:10.1093/nar/gku316 (2014).

4 Kawabata, T. HOMCOS: an updated server to search and model complex 3D structures. *Journal of structural and functional genomics*, doi:10.1007/s10969-016-9208-y (2016).
